# The relative importance of key meteorological factors affecting numbers of mosquito vectors of dengue fever

**DOI:** 10.1371/journal.pntd.0011247

**Published:** 2023-04-13

**Authors:** Yan Liu, Xia Wang, Sanyi Tang, Robert A. Cheke

**Affiliations:** 1 School of Mathematics and Statistics, Shaanxi Normal University, Xi’an, Shaanxi, China; 2 Natural Resources Institute, University of Greenwich at Medway, Chatham Maritime, Chatham, United Kingdom; Huazhong University of Science and Technology Tongji Medical College, CHINA

## Abstract

Although single factors such as rainfall are known to affect the population dynamics of *Aedes albopictus*, the main vector of dengue fever in Eurasia, the synergistic effects of different meteorological factors are not fully understood. To address this topic, we used meteorological data and mosquito-vector association data including Breteau and ovitrap indices in key areas of dengue outbreaks in Guangdong Province, China, to formulate a five-stage mathematical model for *Aedes albopictus* population dynamics by integrating multiple meteorological factors. Unknown parameters were estimated using a genetic algorithm, and the results were analyzed by k-Shape clustering, random forest and grey correlation analysis. In addition, the population density of mosquitoes in 2022 was predicted and used for evaluating the effectiveness of the model. We found that there is spatiotemporal heterogeneity in the effects of temperature and rainfall and their distribution characteristics on the diapause period, the numbers of peaks in mosquito densities in summer and the annual total numbers of adult mosquitoes. Moreover, we identified the key meteorological indicators of the mosquito quantity at each stage and that rainfall (seasonal rainfall and annual total rainfall) was more important than the temperature distribution (seasonal average temperature and temperature index) and the uniformity of rainfall annual distribution (coefficient of variation) for most of the areas studied. The peak rainfall during the summer is the best indicator of mosquito population development. The results provide important theoretical support for the future design of mosquito vector control strategies and early warnings of mosquito-borne diseases.

## Author summary

The study and control of the numbers of *Aedes albopictus*, the main vector of dengue fever in China, is crucial for the prevention and control of this mosquito-borne disease. Due to the major impact of rainfall and temperature on mosquito population sizes, we built a mathematical model based on the life cycle of mosquitoes and meteorological and mosquito vector monitoring data in Guangdong Province. Through this model, we explored the effects of temperature, rainfall and their distribution characteristics on the development of the mosquito population and found that they had different effects on the developing stages of mosquitoes in different periods and different locations. Moreover, according to the established meteorological indicators and their importance ranking, it was found that for most areas of Guangdong Province, rainfall was more important than the temperature distribution and the evenness of rainfall distribution, and that the peak rainfall in summer was the best predictor of mosquito population development. In addition, we also developed a method to utilize previous experience in mosquito control, which together with the above conclusions will be of great help in the design of future mosquito vector control strategies and the prediction and early warning of mosquito-borne diseases.

## Introduction

The warm and humid climate of several coastal provinces in southern China is particularly suitable for the breeding of *Aedes albopictus*, the main vector of dengue fever, which inevitably increases the risk of dengue outbreaks [1,2]. Therefore, the characterization and prediction of the population dynamics of *A*. *albopictus*, especially the analysis of the impact of key climate factors on mosquito numbers (based on proxy values derived from two entomological indices), are of practical significance for the prevention and early warning of dengue outbreaks [3–5].

The life cycle of *A*. *albopictus* is divided into four stages: the eggs, larvae and pupae are aquatic, while adult mosquitoes are terrestrial, but each stage is affected by climatic factors such as temperature and rainfall [6,7]. Experimental studies have found that under a constant temperature of 10°C, *A*. *albopictus* could not develop at all, and eggs failed to develop at 15°C, but with the temperature rising to 30°C, the development rate of each stage increased continuously, and the larvae and pupae developed fastest at about 30°C [8]. Moreover, rising temperatures created conditions allowing larvae or adult mosquitoes to overwinter [9–12]. A modelling study found that the quantity of mosquitoes in a specific area was significantly affected by season and the diapause process, and due to the tropical climate and relatively warm winter in Guangzhou, the mosquito numbers there could show single or double peaks [13]. Furthermore, in addition to temperature, the annual total rainfall and rainfall distribution are also important factors that cannot be ignored [14].

The dynamic effects of a single factor on mosquito numbers can be characterized by simple experimental design and model characterization but taking account of the synergistic effect of multiple factors and determining their relative importance is more challenging. Therefore, we constructed a five-stage mosquito population dynamics model (described below) by collecting years of meteorological and mosquito-vector association data in key areas of dengue fever outbreaks in Guangdong Province. The model was then used for exploring the influence of climatic conditions on the development of mosquito populations, to identify the key meteorological indicators affecting mosquito numbers and ranking them in terms of importance. The model was then used to predict mosquito numbers in 2022 and to evaluate the effectiveness of the model and thus provide suggestions for future mosquito vector control strategies and early warnings to assist the control of mosquitoes and diseases.

## Materials and methods

### Data

Guangdong Province is located at 20°13 ’-25°31’ north latitude, 109°39 ’-117°19’ east longitude, located in the southern coastal zone of China, and belongs to the East Asian monsoon region, from north to south, with a subtropical and tropical climate. The province has jurisdiction over 21 prefecture-level cities. Since neighbouring cities share the same meteorological station data, we chose 12 cities to study according to the differences in their meteorological data including Guangzhou, Yangjiang, Shenzhen, Huizhou, Maoming, Shantou, Shanwei, Zhuhai, Heyuan, Meizhou, Zhaoqing and Shaoguan. Daily temperature and rainfall data for 12 municipalities in Guangdong Province during 2015–2021 were obtained from a weather website (rp5.ru), and the Breteau index (BI) and the ovitrap index (MOI), were obtained from the Guangdong Provincial Health Commission (gd.gov.cn).

**Fig 1:**
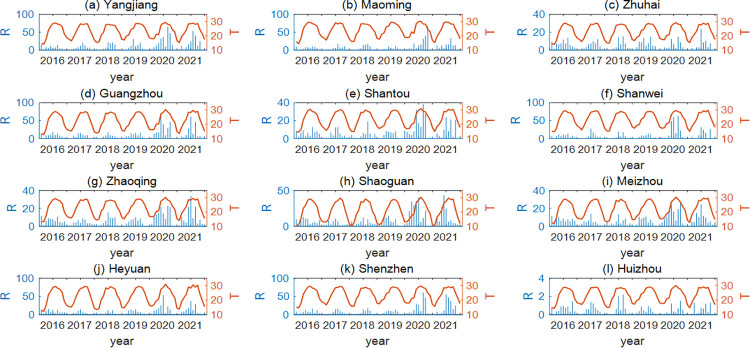
Meteorology data. Average monthly rainfall (blue) and average monthly temperature (red) of 12 cities in Guangdong Province from 2016 to 2021.

The temperature and rainfall data of the 12 cities are shown in Fig 1. It can be seen from the figure that the average monthly temperature is between 10°C and 35°C, with the highest temperatures in June, July, August or September and the lowest temperatures in January or February. The winter of 2019 was the warmest, with the average monthly temperature above 15°C except in Shaoguan. In addition, from the perspective of the monthly average rainfall, most of the 12 cities’ rainfall during 2016–2021 was concentrated in summer, except in 2020 when it was mainly concentrated in summer and autumn. Yangjiang and Guangzhou had the most rainfall, while Huizhou had the least rainfall. Except for Huizhou and Zhuhai, the annual total rainfall showed a trend of low in the first four years and high in the last two years, ranging from 1000mm to 3200mm in the first four years and from 2800mm to 7500mm in the last two years.

### Model

Considering the influence of meteorological factors on each stage of mosquito population growth, we established a stage-structured model. Here, we assume that the mosquito population is large enough in the study area and that the aquatic habitat has enough food for the developing stages of the mosquitoes. The model is as follows:

$$\left\{ \begin{array}{l} \frac{dE_{0}}{dt}=v_{1}(T_{w},W)A-me_{0}E_{0}-de_{0}(t)E_{0}\\ \frac{dE_{d}}{dt}=v_{2}(T_{w},W)A-me_{d}E_{d}-d(T_{w})E_{d}\\ \frac{dL}{dt}=de_{0}(t)E_{0}+d(T_{w})E_{d}-ml(t)L-\frac{ml(t)}{k}L^{2}-dl(t)L\\ \frac{dP}{dt}=dl(t)L-mp(t)P-dp(t)P\\ \frac{dA}{dt}=dp(t)ef(t)P-ma(t)A\\ \frac{dW}{dt}=r(t)-\delta W \end{array}\right.$$ (1)

where *E*_0_ are non-diapause eggs; *E*_*d*_ are diapause eggs; *L* are larvae; *P* are pupae; *A* are adult mosquitoes and *W* is the moisture index. According to Shame et al., the impact of rainfall on the life cycle of mosquitoes is mainly reflected in two aspects: (1) rainfall can increase near-surface humidity, and the increase of humidity will enhance the flight activity and host-seeking behavior of mosquitoes, thus stimulating mosquitoes to lay eggs and accelerate the reproductive cycle of mosquitoes; (2) rainfall can alter the abundance and type of aquatic habitat in which eggs, larvae and pupae live [15]. Therefore, the moisture index *W* associated with rainfall was added into the model to depict the mosquito oviposition rate from the perspective of air humidity, and we described the environmental capacity of the aquatic stage according to rainfall. In the model, as shown in Table 1, *me*_0_ is the mortality rate at the non-diapause egg stage with a value of 0.05 according to the relevant literature [16], and *me*_*d*_ is the mortality rate at the diapause egg stage with value *a* (defined in Table 2) times that of *me*_0_; *r*(*t*) is the total daily rainfall at day *t*; *ef*(*t*) is the emergence rate at day *t*, and *k* is the environmental capacity of the aquatic stages, and they were set according to Tran et al. [16]; *v*_1_(*T*_*w*_,*W*) and *v*_2_(*T*_*w*_,*W*) are the oviposition rates of non-diapause and diapause eggs produced by adult mosquitoes at day *t* respectively, and see below in Eqs (2)–(4) for their settings, and here *T*_*w*_ is the weekly mean temperature; *de*_0_(*t*), *d*(*T*_*w*_), *dl*(*t*), *dp*(*t*) are the development rates at the stages of non-diapause eggs, diapause eggs, larvae and pupae at day *t* respectively, and *ml*(*t*), *mp*(*t*), *ma*(*t*) are the mortality rates at the stages of larvae, pupae and adults at day *t*, respectively, and these parameters were set according to Wang et al. [14]. *δ* is the evaporation rate, shown in Table 2. In addition, the conditions related to the diapause period were set as follows: (1) when the weekly average temperature was lower than 21°C, adult mosquitoes produced diapause eggs; (2) when the weekly average temperature was higher than 15°C, diapause eggs hatched; (3) when the diapause rate was higher than 0.9, the diapause stage began, and the diapause stage ended when diapause eggs hatched (see Supplementary Information for details) [8,16–20]. Therefore, in the model,

$$v_{1}(T_{w},W)=\{\begin{array}{l} 0\;if\;T_{w}<21{}^{\circ}C\\ v(W)\;if\;T_{w}\geq 21{}^{\circ}C \end{array};d(T_{w})=\{\begin{array}{l} 0\;if\;T_{w}\leq 15{}^{\circ}C\\ b\cdot de_{0}(t)\;if\;T_{w}>15{}^{\circ}C \end{array};$$ (2)

See Eq (5) for the expression of *v*(*W*) and see Table 2 for the definition of *b*. Furthermore, according to the latitude of the 12 cities, adult mosquitoes in Yangjiang, Maoming and Zhuhai laid diapause eggs during the diapause period, while no diapause eggs were produced in other cities[21]. Therefore, in Yangjiang, Maoming and Zhuhai,

$$v_{2}(T_{w},W)=\{\begin{array}{l} 0\;if\;T_{w}\geq 21{}^{\circ}C\\ v(W)\;if\;T_{w}<21{}^{\circ}C \end{array},$$ (3)

and in other cities,

$$v_{2}(T_{w},W)=\{\begin{array}{l} 0\;if\;T_{w}\geq 21{}^{\circ}C\;\mathrm{or}\;\mathrm{during}\;\mathrm{diapause}\;\mathrm{period}\;\mathrm{when}\;T_{w}<21{}^{\circ}C\\ v(W)\;if\;T_{w}<21{}^{\circ}C\;\mathrm{and}\;\mathrm{during}\;\mathrm{the}\;\mathrm{non}‐\mathrm{diapause}\;\mathrm{period} \end{array}.$$ (4)

**Table 1:** Parameter definitions.

Parameter	Definition
*ef*(*t*)	The emergence rate
*r*(*t*)	The total daily rainfall
*k*	The environmental capacity of the aquatic stages
*v*_1_(*T*_*w*_,*W*)	The oviposition rate of non-diapause eggs produced by adult mosquitoes
*v*_2_(*T*_*w*_,*W*)	The oviposition rate of diapause eggs produced by adult mosquitoes
*de*_0_(*t*)	The development rate of non-diapause eggs
*d*(*T*_*w*_)	The development rate of diapause eggs
*dl*(*t*)	The development rate of larvae
*dp*(*t*)	The development rate of pupae
*me*_0_	The mortality rate of non-diapause eggs
*me*_*d*_	The mortality rate of diapause eggs
*ml*(*t*)	The mortality rate of larvae
*mp*(*t*)	The mortality rate of pupae
*ma*(*t*)	The mortality rate of adults

Besides, the expressions for *v*(*W*), the development rate, the mortality rate, the emergence rate and the environmental capacity in the model are set as follows:

$$\left\{ \begin{array}{l} v(W)=b_{0}+\frac{E_{\max}}{1+\exp\left[-\frac{\left(W(t)-E_{mean}\right)}{E_{\mathrm{var}}}\right]};\\ dX(t)=A_{X}\frac{T(t)+273.15}{298.15}\frac{\exp\left[\frac{HA_{X}}{1.987}(\frac{1}{298.15}-\frac{1}{T(t)+273.15})\right]}{1+\exp\left[\frac{HH_{X}}{1.987}(\frac{1}{TH_{X}}-\frac{1}{T(t)+273.15})\right]},X\in \{e_{0},l,p\};\\ mY(t)=1-\mu_{Y}\exp\left[-\frac{{\left(T(t)-T_{Y}\right)}^{2}}{V_{Y}^{2}}\right],Y\in \{l,p,a\};\\ ef(t)=\exp\left[-0.1(1+\frac{P(t)}{k(t)})\right];k(t)=250000(R_{norm}(t)+1); \end{array}\right.$$ (5)

where *T*(*t*) is the daily mean temperature; *R*_*norm*_(*t*) represents the normalized value of the two-week total rainfall. Other parameters in Eq (5) and the references for upper and lower bound values about these parameters are shown in Table 2.

**Table 2:** Parameter definitions.

Parameter	Definition	References for upper and lower bound values
*b*_0_	Baseline oviposition rate	[22]
*E*_max_	Maximum oviposition rate	
*E*_*mean*_	The *W*(*t*) value when the oviposition rate is 0.5 *E*_max_	
*E*_var_	Variance	
$A_{X},X\in \{e_{0},l,p\}$	The development rate in the absence of temperature inactivation of key developmental enzymes	[23], [24]
$HA_{X},X\in \{e_{0},l,p\}$	The enthalpy of activation of enzyme-catalyzed reactions	
$HH_{X},X\in \{e_{0},l,p\}$	The change in enthalpy associated with high temperature inactivation of enzymes	
$TH_{X},X\in \{e_{0},l,p\}$	The temperature at which 50% of the enzyme is inactivated by high temperature	
$\mu_{Y},Y\in \{l,p,a\}$	The survival rate at *T*_*Y*_	[14], [22], [25], [26]
$T_{Y},Y\in \{l,p,a\}$	The optimum survival temperature	
$V_{Y},Y\in \{l,p,a\}$	Variance	
***a***	The ratio of mortality rate of diapause to non-diapause eggs	[13]
***b***	The ratio of developmental rate of diapause to non-diapause eggs	
***δ***	The evaporation rate	[14]

### Methods

See Tables 1 and 2 and Eqs (2)–(5) for the meaning and expression of variables in the model. The meanings of 28 parameters estimated in this paper are shown in Table 2, and the parameter estimation results are shown in the Supplementary Information (S1 Table). Due to the large number of selected cities, before fitting, we first classified the 12 cities according to the insects’ overwintering mode as the main basis, supported as an auxillary by differences between the meteorological data. Thus, Yangjiang, Maoming and Zhuhai were placed in the first category; Guangzhou, Shantou, Shanwei, Zhaoqing, Shaoguan, Meizhou and Heyuan in the second category; Shenzhen in the third category; and Huizhou in the fourth category. Then, four groups of parameters were obtained by parameter estimation in Yangjiang, Guangzhou, Shenzhen and Huizhou selected as the "fitting cities" for fitting data to. All cities in each category shared a set of parameters, and the simulation results of the remaining cities except the fitting city were used for model verification. The analyses below under the four sets of parameters were carried out in the four fitting cities (Yangjiang, Guangzhou, Shenzhen and Huizhou).

For regional parameter estimations, 12 cities were classified. Firstly, according to the literature and the latitude of the 12 cities, in Yangjiang, Maoming and Zhuhai, eggs overwintered mainly, followed by larvae, while in other cities it is the overwintering of diapause eggs [21]. In addition, there is little difference in temperature distribution among the 12 cities (Fig 1), so only the differences in rainfall data are considered in the classifications. Here, we used the rotating empirical orthogonal function method to classify the rainfall data of the 12 cities from 2015 to 2021 by region [27,28]. The 12 cities were finally divided into four categories by combining the two factors. When fitting parameters in each category, the sum of the Euclidean distances between the monthly relative numbers of BI and MOI and the monthly relative numbers of larvae and adult mosquitoes were used for the objective function, and then a genetic algorithm was used to find the optimal parameters [29].

In order to explore the key meteorological factors affecting the annual development of mosquito populations and rank their importance, we first clustered the 84 years’ data of the 12 cities from 2015 to 2021 by year using k-Shape clustering to extract the typical climate distribution, and finally the rainfall and temperature were divided into six categories [30]. Then we took the centroid of each category as the typical climate distribution. Combined with the annual total rainfall (1500mm, 2500mm, 3500mm, 5500mm, 7500mm, 9500mm), we arranged and combined the three meteorological variables (typical temperature distribution, typical rainfall distribution, annual total rainfall), and finally brought them into the model for simulation to explore the key meteorological factors affecting the development of mosquito populations throughout the year and indexed them.

We next used the random forest method to rank the importance of 11 meteorological indicators, and then used grey correlation analysis to verify the ranking results [31–33]. Due to the low risk of dengue fever outbreaks in winter in Guangdong Province, the government only publishes the mosquito vector monitoring results from March to October. Therefore, in order to maintain the comparability between subsequent verification results and random forest results, the total number of adult mosquitoes from March to October was selected as the dependent variable, and 11 meteorological indicators were used as independent variables. Based on the data of 84 years from 2015 to 2021 in the 12 cities, the importance of indicators was ranked by random forest. Then only 11 meteorological indicators in 2016, 2017 and 2019 were used for grey correlation analysis on the adult mosquito monitoring index (MOI) because the publication frequency of MOI in 2015 was inconsistent with those in 2016–2021, while MOI data in 2018 were too sparse, and the MOIs for 2020 and 2021 were affected by COVID-19. The indices were ranked according to the magnitude of correlation degree to verify the ranking results based on the random forest analyses.

Finally, in order to draw on the experience of mosquito control practice in previous years, we used the grey correlation analysis method again to analyze the meteorological indicators of each city from 2016 to 2022, taking the 11 meteorological indicators in 2022 as the reference sequence, the meteorological indicators of 2016 to 2021 as the comparison sequence, and the normalized results of the previously calculated grey correlation degree as weighted meteorological indicators. The grey correlation degree between meteorological indicators in 2016–2021 and 2022 was calculated. The greater the correlation degree, the more similar the climate distribution in a year was to that in 2022, and the closer the mosquito population development was without considering the influence of other factors.

## Results

### 1 Estimation and fitting

**Fig 2:**
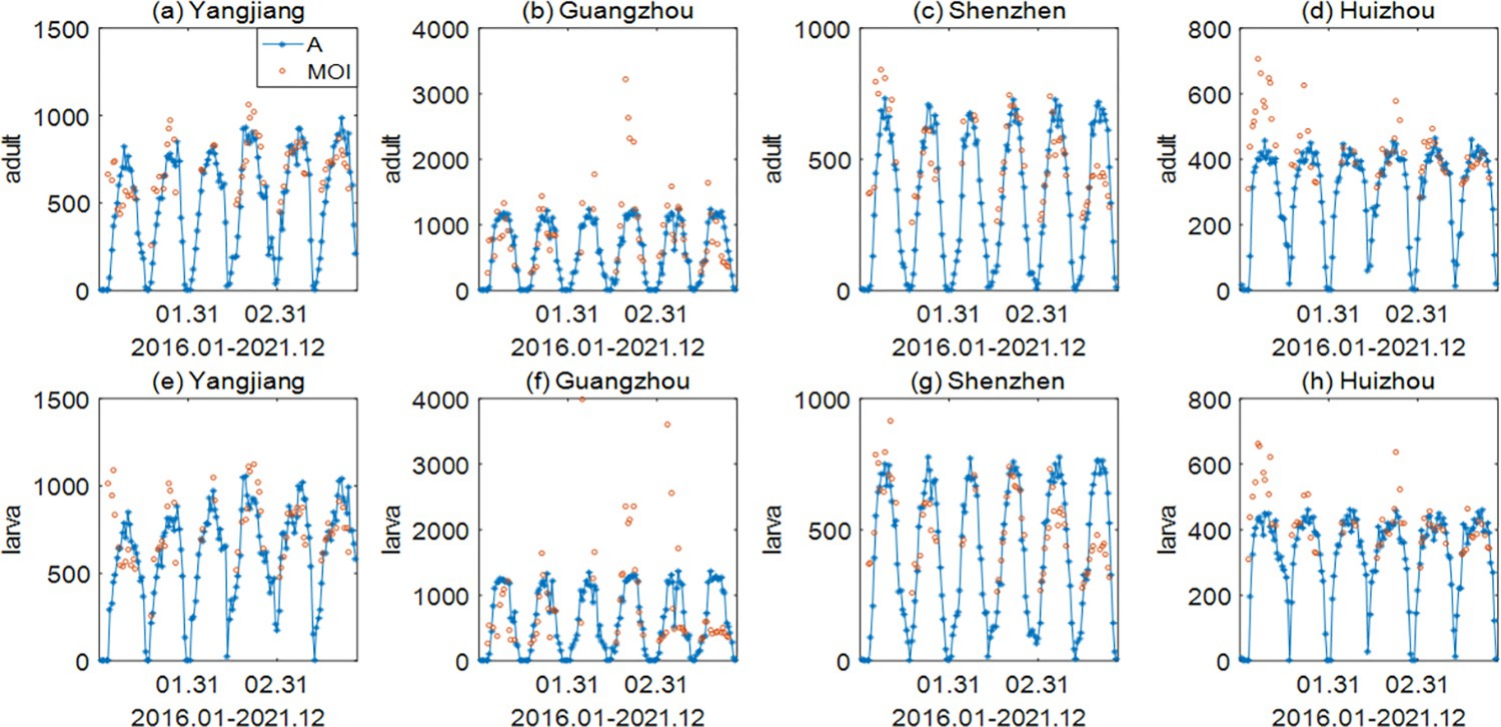
Model fitting results for Yangjiang, Guangzhou, Shenzhen and Huizhou. (a)-(d) Fitting results for adult mosquitoes (MOI); (e)-(h) Fitting results for larvae (BI). Red circles represent the actual MOI and BI values. The blue asterisks represent the compressed fitting result. In order to clearly show the consistency of the changing trend in the simulation results and monitoring indicators in the figure, we compressed the simulation results.

The model fitting results are shown in Fig 2, and the verification results are shown in the Supplementary Information (S1 and S2 Figs). According to the monitoring method published by the Guangzhou Center for Disease Control and Prevention, staff check the monitoring sites every four days, collect positive containers and add new containers in their place (gzcdc.org.cn). In addition, there are enough monitoring sites set in every city, so BI and MOI can reflect the change in trends of mosquito numbers, which is proportional to the actual mosquito population. Therefore, the goal of parameter estimation is that the fitted trends of adult mosquitoes and larvae are consistent with the change in trends of BI and MOI. Fig 2 shows that the fitted trends of adult mosquitoes and larvae in Yangjiang, Guangzhou, Shenzhen and Huizhou from 2016 to 2021 are mostly consistent with the actual MOI and BI trends, but there were overestimates for Guangzhou and Shenzhen in 2021, which may be related to the epidemic and control of COVID-19 in the past two years. Secondly, according to the Guangdong Provincial Health Commission, the monitoring efforts of BI and MOI are different in different cities and years, so the data have a certain degree of randomness and uncertainty, which leads to the overestimation in Yangjiang in 2016 and the underestimation in Huizhou in 2016 and Guangzhou in 2019. In addition, the implementation strength of mosquito control policies by the health commission is different in each city. Also, the BI and MOI are affected by mosquito control policies and monitoring efforts, so the validation results in some years will be poor due to the different implementation strengths of policies and monitoring efforts of BI and MOI in different cities. For example, no policy was implemented in Maoming in 2016, so there was an underestimate when the estimated parameters of Yangjiang were used to fit the mosquito numbers of Maoming. Moreover, the difference in the implementation strength of policies between the fitting city and the validating city also led to the overestimation phenomenon of Zhuhai in 2019 and the underestimation phenomenon of Shantou, Zhaoqing, Shaoguan and Meizhou in 2016. Ignoring these cases and the impact of COVID-19 in 2020 and 2021, it can be seen that the simulated trends for the 8 cities within 6 years are basically consistent with the actual MOI and BI trends under their respective parameters in the Supplementary Information (S1 and S2 Figs), which indicates that the model fitting results are basically consistent with the actual situation. Based on the parameter values estimated above, we can calculate the diapause duration of each city each year, and then find that adult mosquitoes and larvae overwinter in years with a diapause period less than 30 days by comparing the diapause duration with the fitted results of each city (S3 and S4 Figs).

### 2 Factor analyses: Temperature and rainfall

**Fig 3:**
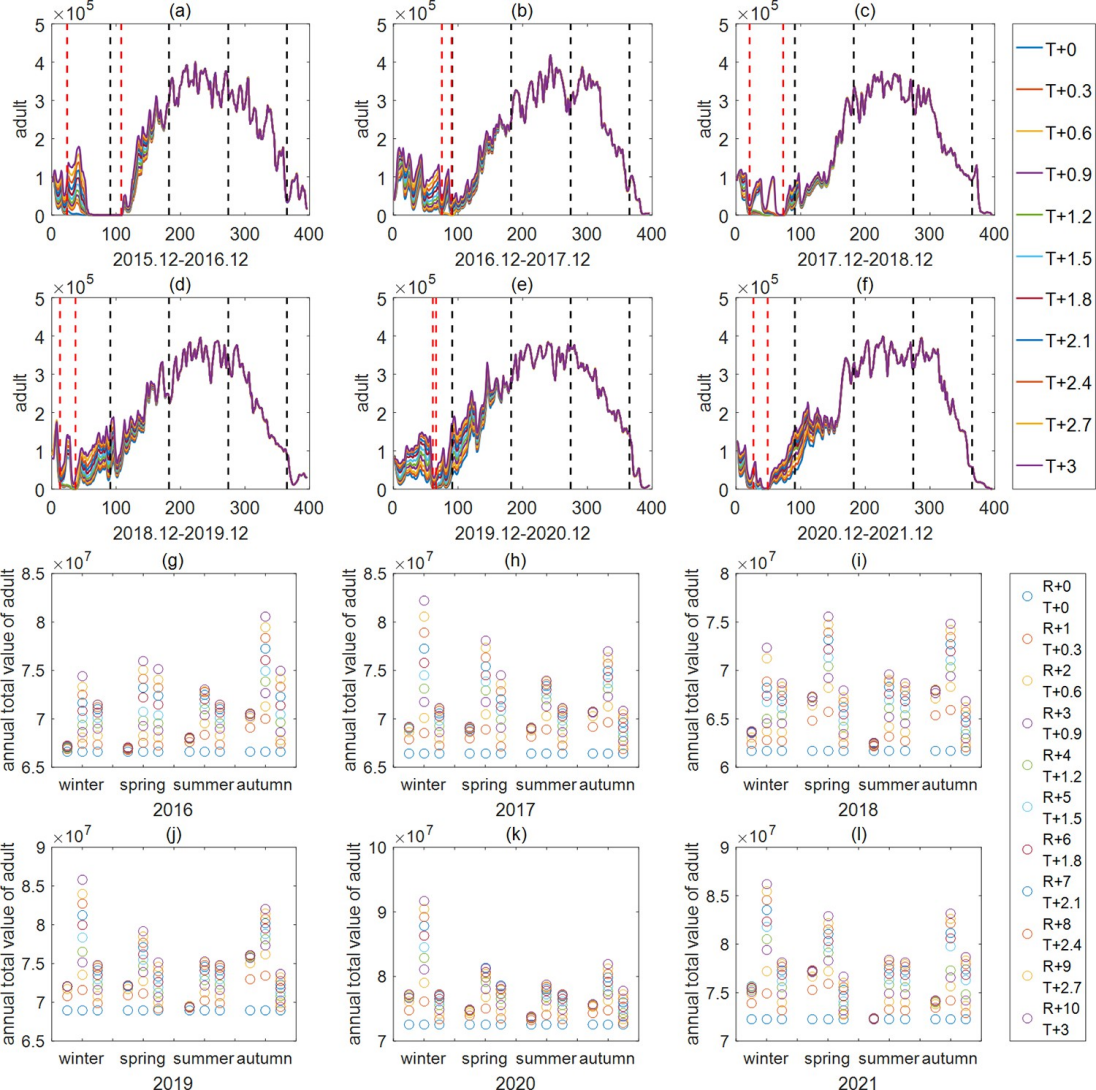
Results of sensitivity analysis in Shenzhen from 2016 to 2021. (a)-(f) Variation of adult mosquito quantity with increasing winter temperature in Shenzhen. The red dashed line from left to right represents the start and end times of diapause, and the black dashed line from left to right represents the ends of winter, spring, summer and autumn. (g)-(l) Effects of seasonal climate change on annual total adult mosquito quantity in Shenzhen. The three sets of data for each season in the subfigure show the results of increasing seasonal rainfall, increasing both seasonal rainfall and seasonal temperature, and increasing seasonal temperature from left to right. The "winter" in figure (g)-(l) represents the winter of the previous year. For example, "winter" in figure (g) represents the winter of 2015, including December 2015 and January and February 2016.

For exploring the influence of temperature and rainfall on the diapause period, double peaks of mosquito numbers in summer and annual total adult mosquito quantities, sensitivity analysis was carried out in the four fitting cities (Yangjiang, Guangzhou, Shenzhen and Huizhou) under four sets of parameters. Three different scenarios were simulated for each year and each season from 2016 to 2021 (2015 was omitted to avoid the impact of initial values): (a) increased seasonal rainfall; (b) simultaneously increased seasonal rainfall and temperature; (c) increased seasonal temperature. In the case of Shenzhen (Fig 3, the sensitivity analysis results in Yangjiang, Guangzhou and Huizhou are shown in the Supplementary Information (S5–S7 Figs)), the results reveal that increasing seasonal temperatures, seasonal rainfall or both can prolong the impacts on adult mosquito numbers. The aftermath of the impact of changing weather conditions in spring and summer can last for 1 to 2 months, while it can last for 1 to 6 months in winter and autumn (Table 3). However, the aftermath duration in Huizhou is significantly shorter than that in the other three cities, which may be related to the low rainfall in that city in previous years. In addition, as can be seen from the actual MOI in the Supplementary Information (S1 Fig), the adult mosquito numbers in Guangzhou, Shantou, Zhaoqing and other cities show double peaks in summer. According to the sensitivity analysis for these cities, the bimodal phenomenon will disappear with increasing summer rainfall, will strengthen with increasing summer temperature, and weaken when the two increase at the same time (S8–S12 Figs), which indicates that the bimodal phenomenon is mostly related to rainfall. By comparing the monthly average rainfall of the corresponding months in these years, it was found that when the monthly rainfall declines steeply, followed by a rise, the double peak phenomenon is highly likely. This condition occurs in summer and the greater the difference between early and late summer rainfall, the more pronounced is the double peak. For instance in the summer of 2020, the MOI data show that there was a double peak in Guangzhou, where the actual monthly average rainfall in each month from May to September was 68.15mm, 40.08mm, 8.61mm, 28.29mm and 46.03mm, respectively, which is more obvious than that in Zhaoqing, where the actual monthly average rainfall from May to August was 21.13mm, 14.3mm, 8.05mm and 22.58mm, respectively. This suggests that a sudden drop of mosquito numbers in one month during the summer could be followed by a new increase or outbreak.

**Table 3:** The aftermath durations of effects of seasonal climate change.

	Yangjiang	Guangzhou	Shenzhen	Huizhou
**Winter**	3 to 5 months	1 to 2 months	2 to 3 months	1 month
**Spring, summer**	2 months	1 month	1 month	1 month
**Autumn**	2 quarters	2 quarters	2 quarters	1 quarter

From Figs 3 and S5–S7, we find that except for Yangjiang in winter 2015 and spring 2018 and 2019, Guangzhou in summer, and Huizhou, increasing seasonal temperature and rainfall both increase the annual total numbers of adult mosquitoes. When both are increased at the same time, the effect on the increase of mosquitoes is the most pronounced. Since the temperature in Guangzhou is mostly between 26°C and 32°C in summer and the development of larvae will be slowed down at this time, as the temperature increases in summer, this situation will be aggravated, and the number of adult mosquitoes will decrease [8,12]. Therefore, increasing temperatures in summer or increases of temperature and rainfall at the same time in Guangzhou do not have a large positive impact on the annual total adult mosquito numbers. The reason for these abnormal results of the sensitivity analysis in spring of Yangjiang in 2018 and 2019 is shown in the Supplementary Information (S13(A) Fig), and the reason for the abnormal results in Huizhou are probably related to the low rainfall. In addition, although increasing winter temperature increases the mosquito development rate, it shortens the diapause period, and a short diapause period will lead to less accumulation of diapause eggs during this period than if the diapause period was normal. Thus, this will result in a decrease in the adult mosquito numbers in the following spring within a certain range (S13(B) Fig), which will in turn lead to a decrease in the adult mosquito quantity throughout the year if the climate remains unchanged in other seasons. For example, the annual total adult mosquito quantity in Yangjiang decreased significantly when the temperature increased in the winter of 2015 (S5 Fig). So, the mild winters may weaken the diapause’s protective effect on mosquito populations and only have a positive effect when the winter is warm enough [11].

### 3 Key factors and their order

**Fig 4:**
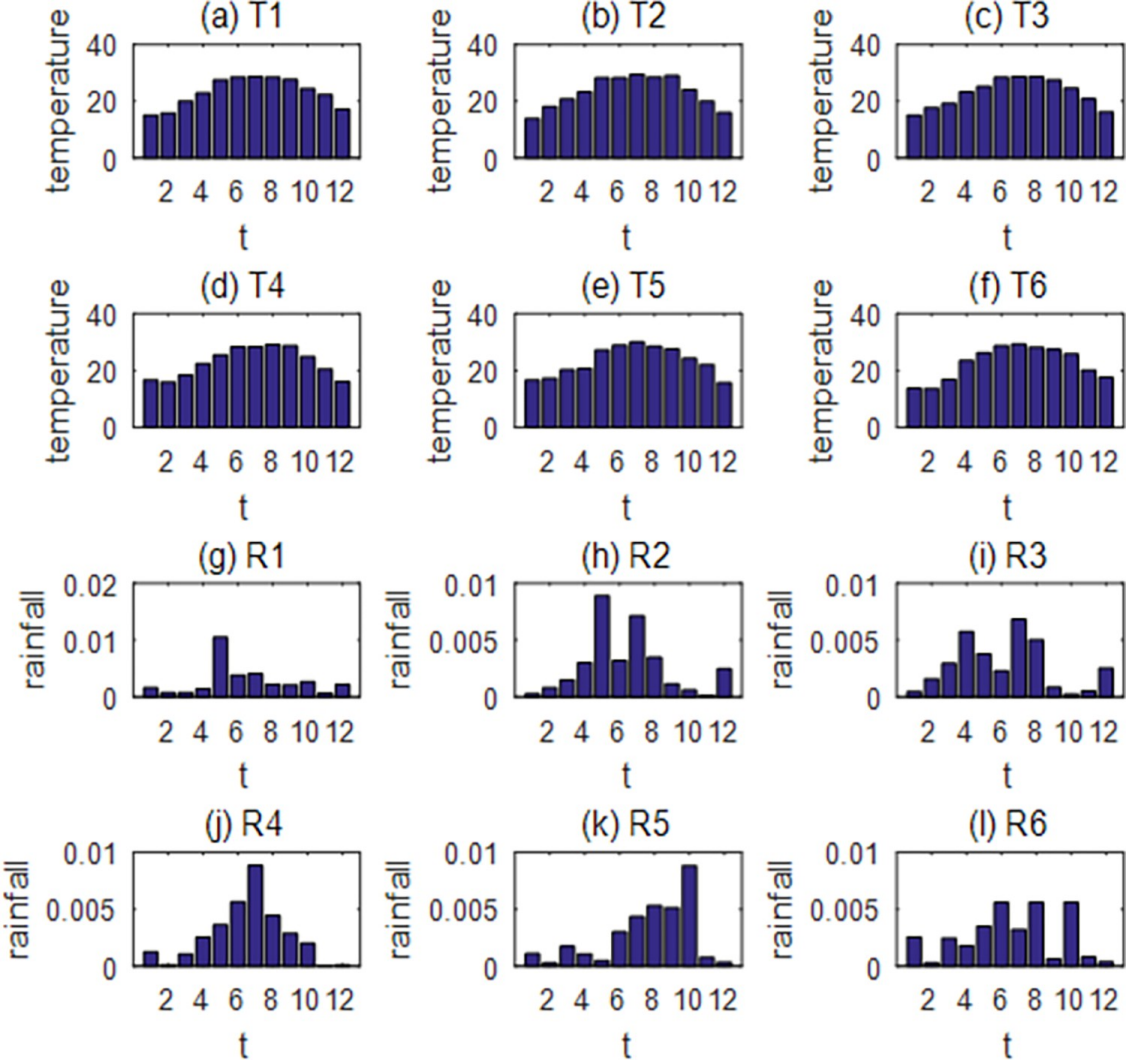
The k-Shape classification results of rainfall and temperature in 84 years. The horizontal coordinate represents the month, and the vertical coordinate represents the monthly mean temperature in (a)-(f) and the monthly total rainfall after normalization in (g)-(l).

In order to further explore the important meteorological factors affecting the development of mosquito populations throughout the year, the temperature and rainfall data in 12 cities from 2015 to 2021 for 84 years were clustered according to the annual distribution shape by the k-Shape method. Through calculation, it was found that the Silhouette Coefficient was the highest (0.6150) when the temperature was classified into six categories. For rainfall, although the Silhouette Coefficient was the highest when rainfall was classified into eight categories (S2 Table), it was found from the centroid distribution that the six categories could cover all rainfall distribution situations (S14–S16 Figs). Therefore, considering the Silhouette Coefficient and classification purpose, the temperature was eventually classified into 6 categories, denoted as T1, T2, T3, T4, T5, T6 (Fig 4(A)-4(F)), and the rainfall was eventually classified into 6 categories, denoted as R1, R2, R3, R4, R5, R6 (Fig 4(G)-4(L)).

As can be seen from Fig 4, the distribution characteristics of R1, R4 and R5 are respectively that the rainfall peak is in spring, summer and autumn; the distribution characteristics of R2 and R3 are double peaks and the distribution of R6 is more uniform than those of the others. There is no obvious difference in temperature distribution in the figure, and the peaks are all in summer, with the coldest temperatures in winter. According to the experimental results of Li et al., the suitable survival temperature range of mosquitoes is 25°C-35°C, and when the temperature is lower than 15°C, the eggs will not hatch [8]. Therefore, the difference between the number of days between 25°C-35°C and the number of days below 15°C in the temperature distribution is used in the following to depict the characteristics of the temperature distribution, denoting it as the temperature index.

**Fig 5:**
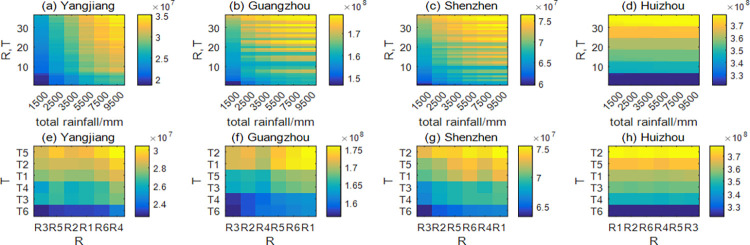
The change of annual total adult mosquito numbers under different rainfall and temperature distribution combinations. The color in the figure represents the annual total adult mosquito numbers. The yellower the color, the higher the annual total adult mosquito numbers. (a)-(d) Variation of annual total adult mosquito quantity with annual total rainfall under the same combination of rainfall and temperature distribution. The y-axis label "R, T" represents the combinations of rainfall distributions (R1, R2, R3, R4, R5, R6) and temperature distributions (T1, T2, T3, T4, T5, T6), such as R1 and T1, R2 and T1, R3 and T5, and so on. There are 36 of them, so the y-axis ranges from 1 to 36. (e)-(h) Variation of annual total adult mosquito quantity when the combination of rainfall and temperature distribution is different under the same annual total rainfall (3500mm). In other words, (e)-(h) are representations of the third column in (a)-(d), respectively.

Then, the annual total rainfall was divided into six grades: 1500mm, 2500mm, 3500mm, 5500mm, 7500mm and 9500mm. Under four sets of parameters, namely, in Yangjiang, Guangzhou, Shenzhen and Huizhou, the changes of annual total adult mosquito quantity under different rainfall and temperature distribution combinations were explored. Except for Huizhou, the results indicate that the annual total adult mosquito number increases with increasing annual total rainfall when the distributions of rainfall are the same and the distributions of temperature also are the same. For example, when the distributions of rainfall and temperature in Yangjiang are R4 and T5, with the increase of annual total rainfall, the annual total adult mosquito quantity is 2.5219e+07, 2.8226e+07, 3.0438e+07, 3.4180e+07 and 3.5165e+07 in turn (Fig 5(A)). Moreover, when the annual total rainfall amounts are the same (3500mm), the annual total adult mosquito quantity varies with temperature and rainfall distributions: the number of mosquitoes in Yangjiang is the highest (3.0438e+07) under combination R4 and T5, and the lowest (2.2586e+07) under combination R3 and T6, with a difference of 35% (Fig 5(E)). Besides, due to the low rainfall in Huizhou, the meteorological conditions are significantly different from the other three cities, the total annual rainfall and rainfall distribution in Huizhou have almost no influence on the annual total adult mosquito quantity, which is mainly related to the temperature distribution (Fig 5(D) and 5(H)).

**Fig 6:**
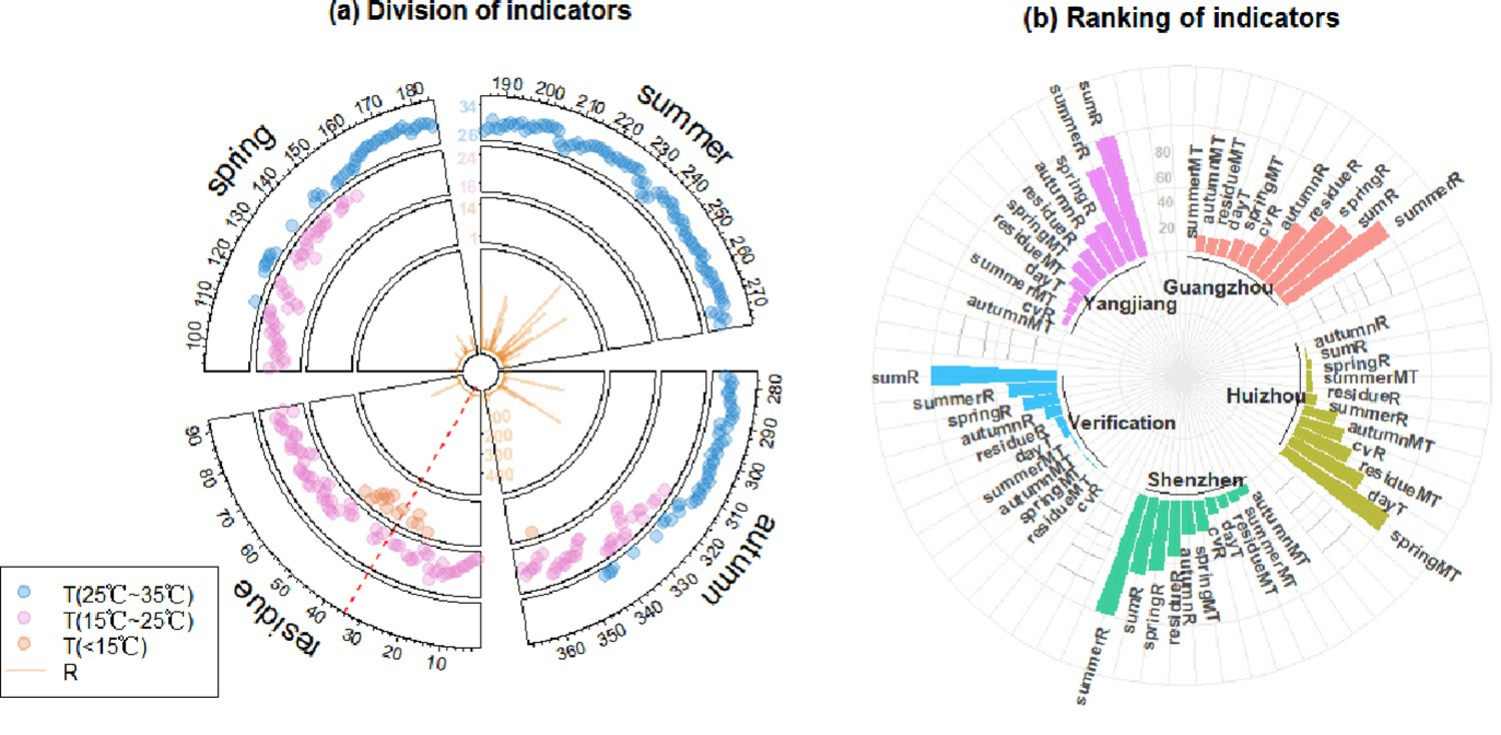
Division and importance rank of meteorological indicators. (a) Taking Shenzhen in 2021 as an example, the figure shows the division of meteorological indicators within a year, calculated using the *circlize* package in R [35]. The red dotted line in the figure represents 1 January, the "residue" includes January, February and December in a year, and a sector represents a season. From the outside to the inside, the first three rings together show the changes of daily mean temperature on 365 days in a year clockwise, among which the data values are in the range of 25°C-35°C, 15°C-25°C, and less than 15°C, respectively. The fourth ring shows the changes of daily total rainfall in a year clockwise. The mean temperature and total rainfall in the four sectors are denoted as *residueMT*, *springMT*, *summerMT*, *autumnMT*, *residueR*, *springR*, *summerR* and *autumnR*. In addition, the coefficient of variation (*cvR*) of rainfall data is used to represent the uniformity of the rainfall distribution, which is equal to the standard deviation of the annual rainfall data divided by the mean, and the annual total rainfall is denoted *sumR*. The difference between the number of days between 25°C-35°C and less than 15°C is defined as the temperature index (*dayT*), that is, the difference between the number of data points in the first ring band and the third ring band. (b) Importance ranking results of the above meteorological indicators by random forest and verification results of indicators importance ranking.

Furthermore, seasonal rainfall, reflecting the location of peak rainfall, annual total rainfall, seasonal mean temperature that also affects mosquito populations as shown in section 2, the coefficient of variation of rainfall, reflecting the uniformity of the annual distribution of rainfall, temperature index were selected as meteorological indicators (Fig 6(A)). Next, the random forest method was used to rank the importance of the above meteorological indicators. The main results are shown in Fig 6(B). The results show that, except for the fourth category of Huizhou, in the other three categories of cities, the rainfall indicators are more important than the temperature indicators and the coefficient of variation, and the importance of the rainfall peak position in the rainfall indicators follows summer > spring > autumn. This indicates that for these three cities, rainfall is more important than the temperature distribution (spring, summer, autumn, residual average temperature and temperature index) and the uniformity of rainfall annual distribution (coefficient of variation), and the peak rainfall during the summer is the best for mosquito population development [34]. Therefore, for the development of mosquito populations throughout the year, the uniformity of the rainfall distribution is not so important. The peak position is more important, which may be related to the fact that the annual total rainfall of these three cities from 2015 to 2021 is between 1000mm and 7500mm, and that they belong to Guangdong Province and there is no obvious difference in temperature distribution. That is, for neighbouring areas, when the total rainfall reached a certain level, the influence of temperature distribution and evenness of rainfall distribution on mosquito quantity would be weakened. However, for Huizhou, the annual total rainfall from 2015 to 2021 is between 100mm and 300mm, and is very small, so the temperature distribution is more important for the mosquito population size in that city.

To verify the above ranking results, we used the grey correlation analysis method to rank the correlation degree of the above meteorological indicators on MOI (Fig 6(B)). The verification results are basically consistent with the experimental results of the first three types of cities. Likewise, rainfall is the most important indicator, with peak rainfall in summer being the best for year-round mosquito population development. To sum up, the establishment and importance ranking of meteorological indicators in this paper have been shown to be reliable.

### 4 Prediction and possibility of early warning?

**Fig 7:**
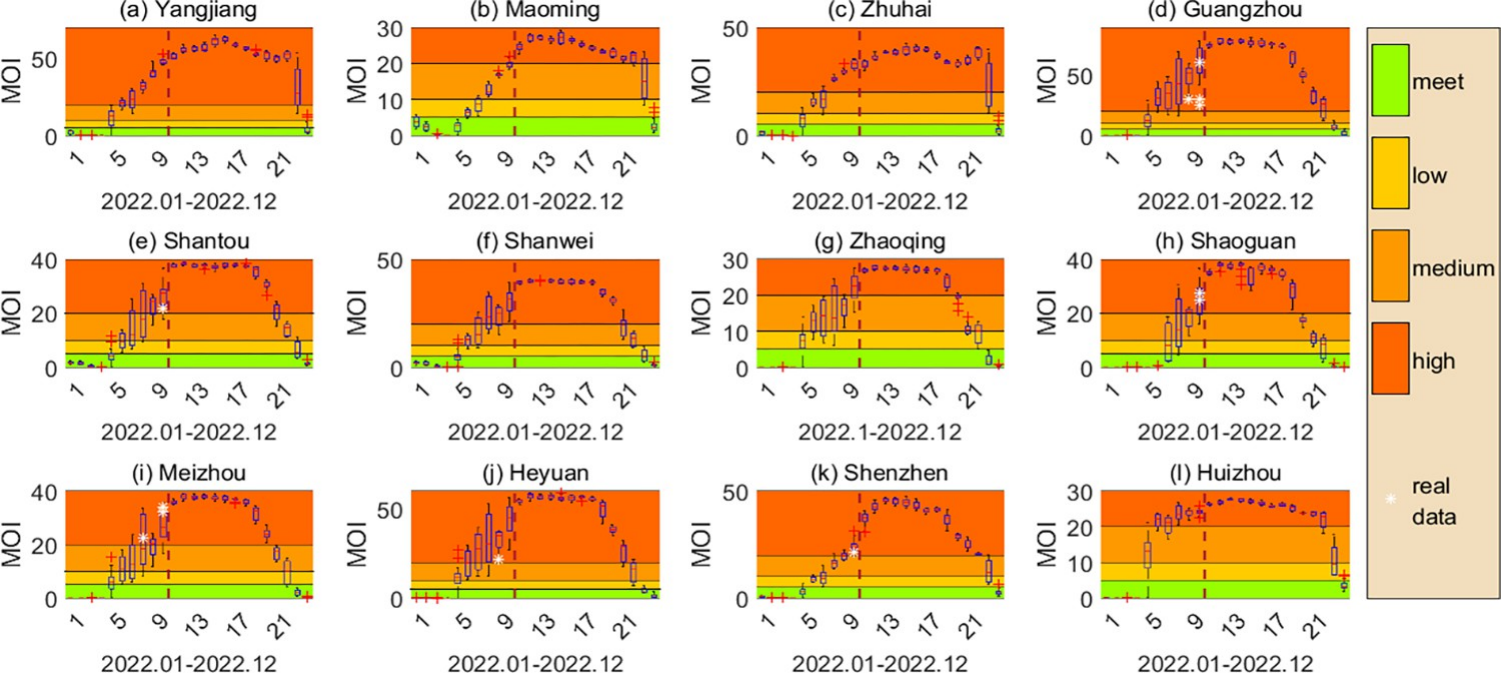
Predicted results for the MOIs for 12 cities in Guangdong Province in 2022. Different colour bands represent different risk levels which are "meet the prevention and control requirements" (MOI<5), "low risk level" (5<MOI<10), "medium risk level" (10<MOI<20) and "high risk level" (MOI>20) from bottom to top, respectively. The left side of the dotted line is January-May in which the MOI is predicted by real weather data, and the right side is June-December in which the MOI is predicted by substituting the mean of the weather data of corresponding months in the previous seven years into the model. The white asterisk in the figure represents the MOI value published on the official website of Guangdong Provincial Health Commission in 2022.

In order to further verify the effectiveness of the method and provide methodological support for early prediction and early warning of mosquito-borne diseases, we used an established model to predict the mosquito population in 2022 in 12 cities, and the adult mosquito surveillance index MOI was selected for analysis. The weather data from January to May in 2022 used were the actual data, and the weather data from June to December were replaced by the average of the previous 7 years’ data for each city, so as to get the weather data for the whole year, which can be put into the model to calculate the number of mosquitoes in 2022. Since the classification of the risk level about the mosquito quantity in Guangdong Province is based on the monitoring indicators MOI and BI which are published every half month, a box chart is used to display the risk level of the mosquito numbers in each period of 2022 by the unit of half a month. As shown in Fig 7, the cities that have announced MOI monitoring results include Guangzhou, Shantou, Shaoguan, Meizhou, Heyuan and Shenzhen. Except for Guangzhou in which the prediction accuracy is affected by changes in the mosquito vector control measures [36], the other monitoring results are all within the prediction range, which verifies the effectiveness of the model and method.

**Fig 8:**
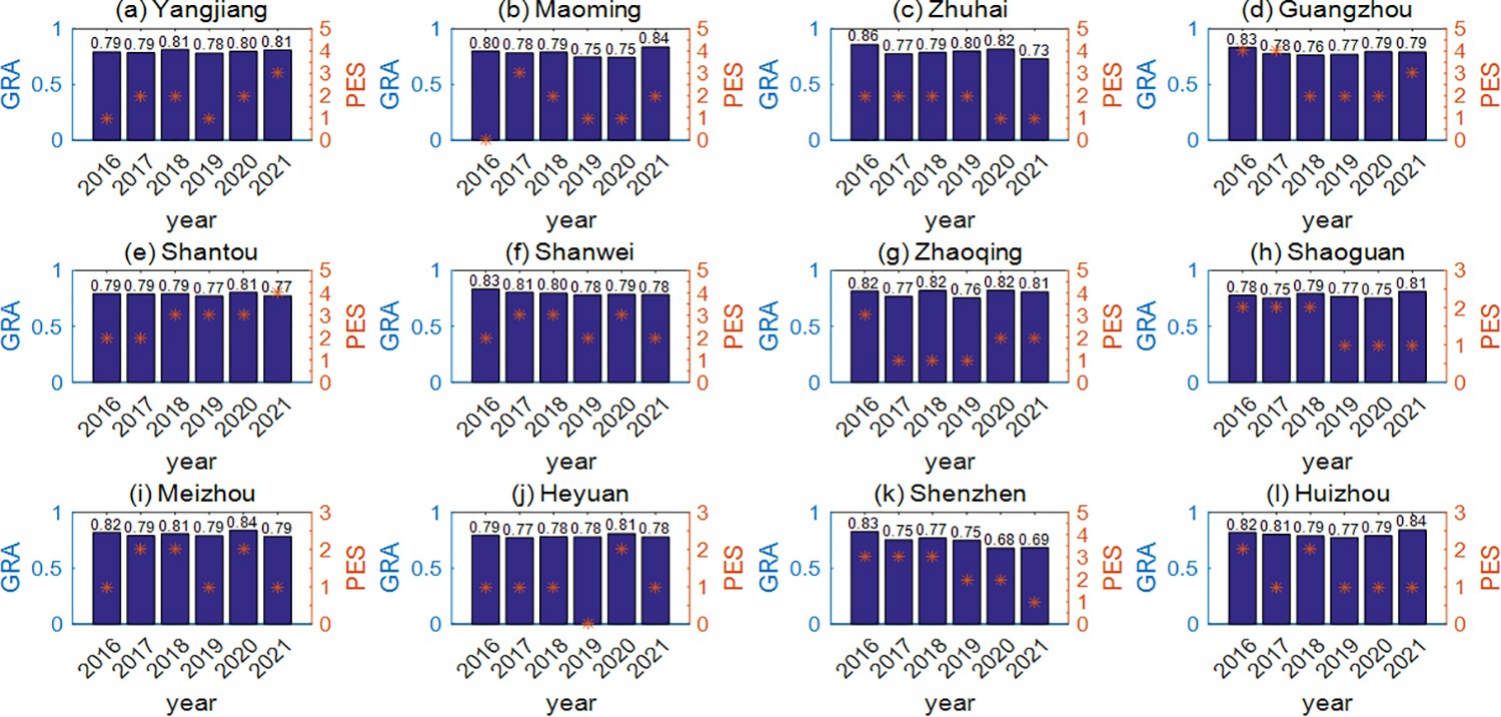
Grey correlation analysis results for 12 cities from 2016 to 2022. The numbers on top of the bars in the figure are the values of the grey correlation degree (GRA). The asterisks represent the policy implementation strength (PES) of the 12 cities from 2016 to 2021. According to the notice on the official website of each municipal government, there are two kinds of specific mosquito control policies: (1) the special mosquito eradication campaign, and (2) the patriotic health campaign which includes measures such as killing adult mosquitoes and removing standing water. According to the frequency of policy implementation, we divide its enforcement strength into five levels. If no policy has been implemented, the 0 is denoted; if the policy has been implemented for ≤ 3 months, the number is 1; if the policy has been implemented for ≤ 6 months or regional control mosquitoes is frequent, the number is 2; if mosquito control was carried out over 6 months, 3 is denoted; if the mosquitoes were controlled for more than 6 months but also at least twice a month, 4 is denoted.

In order to make use of successful mosquito control experience in previous years, the meteorological indicators from 2016 to 2022 were analyzed by grey correlation analysis (Fig 8). Conclusions are based on the notion that the greater the correlation, the closer the meteorological indicators and the more similar the climate distributions are between that year and 2022, and then the closer the mosquito population development is without considering the influence of other factors. In this way, we can learn from the experience and policy implementation of that year to implement accurate mosquito control in 2022. According to Fig 8, the meteorological indicators of 12 cities in 2022 had the highest correlation with that for 2018 or 2021 in Yangjiang, 2021 in Maoming, 2016 in Zhuhai, 2016 in Guangzhou, 2020 in Shantou, 2016 in Shanwei, 2016 or 2018 or 2020 in Zhaoqing, 2021 in Shaoguan, 2020 in Meizhou, 2020 in Heyuan, 2016 in Shenzhen and 2021 in Huizhou respectively, all of which are more than 80%. Therefore, the mosquito control work of the corresponding city in 2022 can be carried out by referring to the mosquito control experience of the above years. In conclusion, for Guangdong Province, the best prevention and control strategies can be formulated by grey correlation analysis based on the indicators established in this paper and the previous experience of mosquito control.

## Discussion

This paper focuses on how to explore the synergistic effect of multiple meteorological factors affecting the number of mosquitoes and determine their relative importance. Firstly, based on the life cycle of mosquitoes, a five-stage mathematical model of the population dynamics of *Aedes albopictus* was established, and a genetic algorithm was used to estimate the parameters in different regions, as shown in Figs 2, S1 and S2. Next, sensitivity analysis was carried out on the influence of climate in different seasons on the number of mosquitoes (Figs 3 and S5–S7), and on this basis, typical climate distribution was further extracted by k-Shape clustering to explore the changes in the number of mosquitoes under different combinations of rainfall and temperature distribution (Figs 4 and 5). Finally, we gave the key meteorological indicators affecting the number of mosquitoes and ranked the importance of the indicators by random forest and verified the ranking results by grey correlation analysis, which is helpful for understanding the mechanism of how meteorological conditions influence the development of mosquito populations. At the end of the paper, the model was used to predict the number of mosquitoes in Guangdong Province in 2022 (Fig 7), which verified the effectiveness of the model and method.

The life cycle of mosquitoes is greatly influenced by meteorological conditions, and the mechanism of its influence has always been a subject of curiosity [3–7,37–41]. Our sensitivity analysis of these effects found that the aftermath duration of the impact of changing climate in different seasons on mosquito numbers was different. The aftermath of changing winter climate lasted for 1–5 months, changing spring and summer climate lasted for 1–2 months, and changing autumn climate lasted for 1–2 quarters (Table 3). Moreover, it was found that increasing seasonal temperature and increasing seasonal rainfall both had positive effects on the annual total adult mosquito quantity except for at Yangjiang in winter 2015 and spring 2018 and 2019, Guangzhou in summer, and Huizhou, and the effect was best when the two factors were combined (Figs 3 and S5–S7). To further explore the mechanism, we indexed and ranked meteorological conditions. The results showed that in most areas of Guangdong province, the rainfall was more important than the temperature distribution (seasonal mean temperature and temperature index) and rainfall distribution uniformity (coefficient of variation), and rainfall peak in summer was better than in spring and autumn for the development of the mosquito population (Fig 6). This extends Wang et al. ’s 2016 and 2019 studies while verifying the conclusion of Wang et al. (2019) that the peak rainfall in summer is the best indicator of large-scale development of mosquito populations [14,34]. Of course, Huizhou here is always an exception, and it is speculated that the reason may be related to the lack of rainfall throughout the year.

A large number of experiments have verified that diapause is a protective mechanism for mosquito population overwintering, which is closely related to internal and external factors [9–12,42–44]. For Guangdong Province, some new and interesting conclusions have been found in this paper. Comparing the fitting results and diapause duration, it was found that adult mosquitoes or larvae overwintered in years with a diapause period less than 30 days (S3 and S4 Figs); In addition, we also found that warm winters may reduce the accumulation of diapause eggs due to the shortening of the diapause period, thereby weakening the protective effect of the diapause period on mosquito populations (S13(B) Fig), which is consistent with Armstrong et al.’s 2017 findings that the overwintering of mosquito populations was influenced by the number of diapause eggs laid in the previous autumn and by overwintering temperatures and conditions that influence the egg mortality [11].

In addition to the above conclusions, in the process of exploring the influence of meteorological conditions on mosquito populations, we further found that the double peak phenomenon of mosquito numbers in summer was mainly related to rainfall, which would disappear with the increase of summer rainfall, strengthen with the increase of summer temperature and weaken when the two increased at the same time (S8–S12 Figs) [13]. Moreover, at the end of this paper, we not only digitized the mosquito control policies of Guangdong Province from 2016 to 2021, but also utilized these mosquito control experiences with the established indicators (Fig 8), which provided new directions and ideas for other scholars’ research.

This study not only confirms the importance of rainfall and temperature in mosquito population development, but also shows that their effects are spatio-temporally heterogenous. Thus, it is necessary to take account of geographical and temporal aspects of dengue outbreak zones when making forecasts. However, the model and parameters are established and determined for the situation of Guangdong Province. Although the model framework and methods can be applied and extended to research in other regions, due to the different mosquito species and climate in different regions, adjustments should be made according to the actual situation, such as estimating MOI and BI through binomial distribution and simulated oviposition frequency, then compare with the actual MOI and BI to adjust the parameters [45]. In addition, there are some limitations of the model. For example, as hypothesized above, the model in this paper was established under the condition of sufficient food in the breeding grounds, and the impact of food availability on the development of the mosquito populations was not considered; Moreover, the description of environmental capacity and mortality in the model also needs to be further refined, such as by including such influences as that excessive rainfall will destroy the breeding grounds and that rainfall will increase the mortality of larvae when triggering egg hatching [46].

## Supporting information

S1 Table The upper and lower bounds of parameters and estimation results. (DOC)

S2 Table The Silhouette Coefficients of rainfall and temperature when they were classified into categories 2–10 by the k-Shape method. (DOC)

S1 Fig Fitted results of adult mosquitoes in 12 cities. Blue asterisks represent fitted results, and the red circles represent the actual MOI values.
(TIF)

S2 Fig Fitted results for larvae in 12 cities. Blue asterisks represent fitted results, and the red circles represent the actual BI values.
(TIF)

S3 Fig The overwintering of adult mosquitoes and larvae. The overwintering of adult mosquitoes and larvae in the 12 municipalities from 2016 to 2021 by enlarging the bottom of the fitted results for larvae and adults in S1 and S2 Figs. Blue asterisks represent fitted results for adult mosquitoes and red triangles represent fitted results for larvae.
(TIF)

S4 Fig Diapause duration in 12 cities from 2015 to 2020. The red dashed line represents 30 days.
(TIF)

S5 Fig Effects of seasonal climate change on annual total adult mosquito quantity in Yangjiang. The three sets of data for each season in the sub-figure show the results of increasing seasonal rainfall, increasing both seasonal rainfall and seasonal temperature, and increasing seasonal temperature from left to right. The "winter" in the figure represents the winter of the previous year. For example, "winter" in the first subfigure represents the winter of 2015, including December 2015 and January and February 2016. The same is true for S6 and S7 Figs.
(TIF)

S6 Fig Effects of seasonal climate change on annual total adult mosquito quantity in Guangzhou. (TIF)

S7 Fig Effects of seasonal climate change on annual total adult mosquito quantity in Huizhou. (TIF)

S8 Fig Variation of adult mosquito quantity with changing summer climate in Guangzhou for 2020. Changes in adult mosquito quantity when (a) increasing summer rainfall, (b) increasing summer temperature, and (c) simultaneously increasing summer rainfall and temperature. The black dashed line from left to right represents the end time of winter, spring, summer and autumn. The same is true for S9–S12 Figs.
(TIF)

S9 Fig Variation of adult mosquito quantity with changing summer climate in Shantou for 2020. (TIF)

S10 Fig Variation of adult mosquito quantity with changing summer climate in Zhaoqing for 2020. (TIF)

S11 Fig Variation of adult mosquito quantity with changing summer climate in Shaoguan for 2020. (TIF)

S12 Fig Variation of adult mosquito quantity with changing summer climate in Meizhou for 2020. (TIF)

S13 Fig Variation of adult mosquito quantity with increasing temperature in Yangjiang. (a) The variation of adult mosquito quantity with increasing temperature in the spring of 2019; (b) The variation of adult mosquito quantity with increasing temperature in the winter of 2015, and the two red dashed lines in subgraph (b) represent, from left to right, the start and end of the diapause period (the longest diapause period in the experiment), and the black dashed line represents the winter end date. On the basis of the actual temperature in each season of each year, the temperature was increased by 3°C at intervals of 0.3. The curve in the figure represents the seasonal variation of the number of mosquitoes when the temperature was increased by 0.3. As the spring temperatures of 2018 and 2019 in Yangjiang are mostly between 18°C and 30°C, and the optimal survival temperatures for larvae, pupae and adult mosquitoes are 28°C, 30°C and 21°C respectively, the curves representing the changes in the number of mosquitoes will cross during the process of temperature increase, as shown in S13(A) Fig above, the crossing of thick and thin lines. As a result, the annual total adult mosquito quantity decreases and then rises with the increase of spring temperatures in 2018 and 2019 as shown in S5 Fig.
(TIF)

S14 Fig Centroid distributions when rainfall data in 84 years were classified into 5 categories (a)-(e) and 7 categories (f)-(l). In the figure, the horizontal axis represents the month, and the vertical axis represents the average monthly rainfall after Z-Score standardization. Obviously, compared with these centroid distributions when classified into 6 categories (S15 Fig), subgraphs (a)-(e) do not include the situation when the annual distribution of rainfall is more uniform (S15(F) Fig). In addition, the rainfall peaks of distribution (i) and (l) in subfigures (f)-(l) are both in summer, and the peak height is about 2. According to the classification purpose (to find the annual distribution of typical climate), the two can obviously be classified into the same category.
(TIF)

S15 Fig Centroid distributions when rainfall data in 84 years were classified into 6 categories. The horizontal axis represents the month, and the vertical axis represents the average monthly rainfall after Z-Score standardization.
(TIF)

S16 Fig Centroid distributions when rainfall data in 84 years were classified into 8 categories. The horizontal axis represents the month, and the vertical axis represents the average monthly rainfall after Z-Score standardization. Compared with S15 Fig (these centroid distributions when classified into 6 categories), it is obvious that (d), (g) and (h) can also be classified into the same category, because the rainfall peaks of the three are in summer and the height is around 2.
(TIF)
