# Supplementary material for: The relative importance of key meteorological factors affecting numbers of mosquito vectors of dengue fever
Source: PLoS Negl Trop Dis. 2023 Apr 13;17(4):e0011247. doi: 10.1371/journal.pntd.0011247 (PMC10128945; doi:10.1371/journal.pntd.0011247)
Supplement: S1 Table — (DOC) [file pntd.0011247.s001.doc]

**S1 Table. The upper and lower bounds of parameters and estimation results.**

| **Parameters** | **Lower bound** | **Upper bound** | **Yangjiang** | **Guangzhou** | **Shenzhen** | **Huizhou** |
| --- | --- | --- | --- | --- | --- | --- |
| $b_{0}$ | 0 | 5 | 3.55 | 4.62 | 2.41 | 4.88 |
| $E_{\max}$ | 0 | 20 | 8.51 | 19.46 | 17.57 | 19.86 |
| $E_{mean}$ | -5 | 5 | 0.23 | 1.59 | 1.16 | -0.28 |
| $E_{\mathrm{var}}$ | 0 | 20 | 10.69 | 0.01 | 1.99 | 1.55 |
| $\mu_{l}$ | 0 | 1 | 0.16 | 0.8 | 0.42 | 0.57 |
| $T_{l}$ | 27 | 29 | 27.92 | 28.98 | 28.94 | 27.28 |
| $V_{l}$ | 5 | 20 | 15.13 | 19.76 | 18.24 | 19.79 |
| $\mu_{p}$ | 0 | 1 | 0.45 | 0.91 | 0.87 | 0.99 |
| $T_{p}$ | 29 | 31 | 29.69 | 29.09 | 30.55 | 29.06 |
| $V_{p}$ | 5 | 20 | 19.23 | 19.88 | 7.86 | 19.91 |
| $\mu_{a}$ | 0.4 | 0.95 | 0.91 | 0.46 | 0.93 | 0.69 |
| $T_{a}$ | 20 | 21 | 20.01 | 20.21 | 20.07 | 20.03 |
| $V_{a}$ | 5 | 20 | 7.34 | 19.65 | 7.61 | 19.99 |
| $A_{e_{0}}$ | 0 | 1 | 0.2 | 0.34 | 0.34 | 0.15 |
| $HA_{e_{0}}$ | 0 | 41192.69 | 6886.92 | 41028.65 | 37420.04 | 13021.83 |
| $HH_{e_{0}}$ | 5954.35 | 150000 | 19729.39 | 60009.86 | 13922.06 | 124741.24 |
| $TH_{e_{0}}$ | 300 | 305 | 301.97 | 304.99 | 300.1 | 304.94 |
| $A_{l}$ | 0 | 0.97 | 0.35 | 0.95 | 0.21 | 0.97 |
| $HA_{l}$ | 26018.51 | 41192.69 | 26824.02 | 27223.99 | 27860.14 | 26625.89 |
| $HH_{l}$ | 45543.49 | 81383.14 | 81141.11 | 48035.37 | 57677.63 | 62780.47 |
| $TH_{l}$ | 291 | 313 | 310.75 | 295.57 | 307.41 | 301.12 |
| $A_{p}$ | 0 | 0.97 | 0.44 | 0.45 | 0.45 | 0.56 |
| $HA_{p}$ | 0 | 19246.42 | 19122.99 | 16371.87 | 14774.25 | 13059.41 |
| $HH_{p}$ | -500000 | 5954.35 | -366277.59 | -256795.7514 | -58513.8 | -462867.03 |
| $TH_{p}$ | 0 | 313 | 39.11 | 163.24 | 264.46 | 41.96 |
| $\delta$ | 0 | 1 | 0.67 | 0.61 | 0.36 | 0.22 |
| $a$ | 0 | 1 | 0.99 | 0.96 | 0.97 | 0.97 |
| $b$ | 0 | 1 | 0.0026 | 0.02 | 0.01 | 0.0006 |

In the table, Yangjiang, Guangzhou, Shenzhen and Huizhou are the first category, second category, third category and fourth category fitting cities, respectively.
