# Supplementary material for: The relative importance of key meteorological factors affecting numbers of mosquito vectors of dengue fever
Source: PLoS Negl Trop Dis. 2023 Apr 13;17(4):e0011247. doi: 10.1371/journal.pntd.0011247 (PMC10128945; doi:10.1371/journal.pntd.0011247)
Supplement: S2 Table — (DOC) [file pntd.0011247.s002.doc]

**S2 Table. The Silhouette Coefficients of rainfall and temperature when they were classified into categories 2-10 by the k-Shape method.**

|  | **2 categories** | **3 categories** | **4 categories** | **5 categories** | **6 categories** | **7 categories** | **8 categories** | **9 categories** | **10 categories** |
| --- | --- | --- | --- | --- | --- | --- | --- | --- | --- |
| **Temperature** | 0.3177 | 0.3742 | 0.4673 | 0.5008 | 0.6150 | 0.5701 | 0.5317 | 0.5791 | 0.5895 |
| **Rainfall** | 0.1264 | 0.1586 | 0.1642 | 0.1781 | 0.1733 | 0.2211 | 0.2447 | 0.1716 | 0.1729 |
