# Supplementary figures and images for: The relative importance of key meteorological factors affecting numbers of mosquito vectors of dengue fever
Source: PLoS Negl Trop Dis. 2023 Apr 13;17(4):e0011247. doi: 10.1371/journal.pntd.0011247 (PMC10128945; doi:10.1371/journal.pntd.0011247)

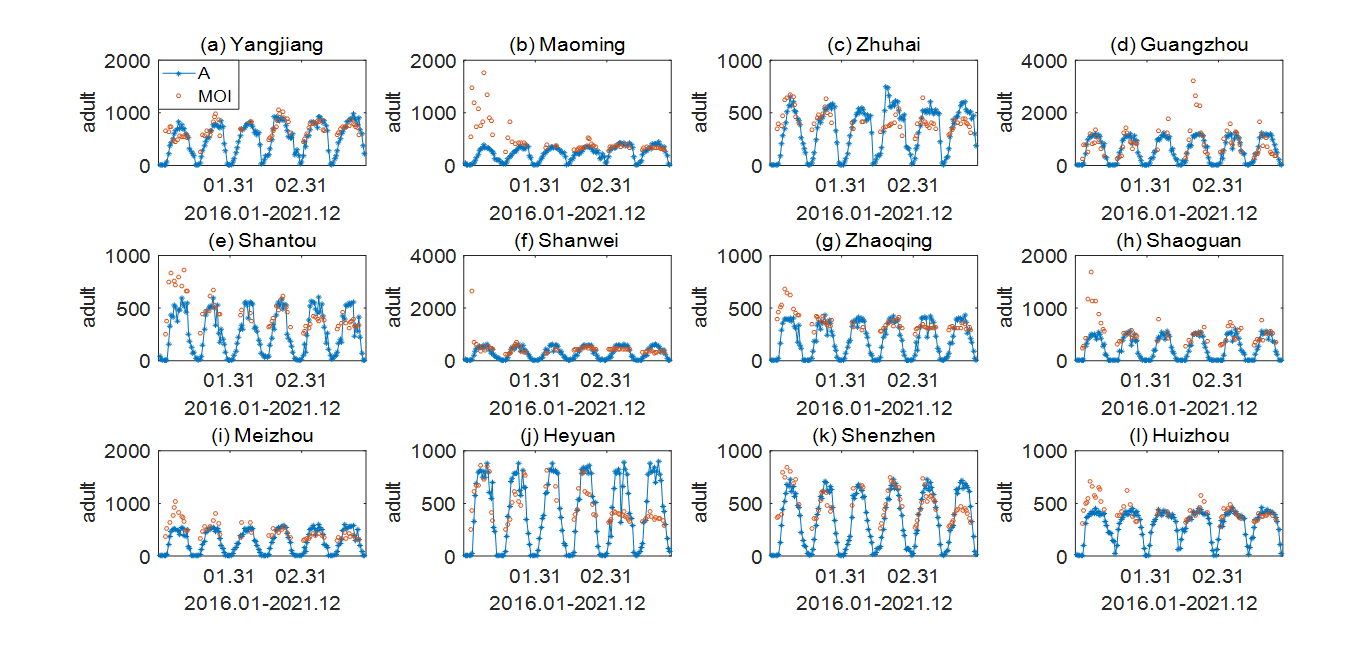

Supplement: S1 Fig — Blue asterisks represent fitted results, and the red circles represent the actual MOI values. (TIF) [file pntd.0011247.s003.tif]

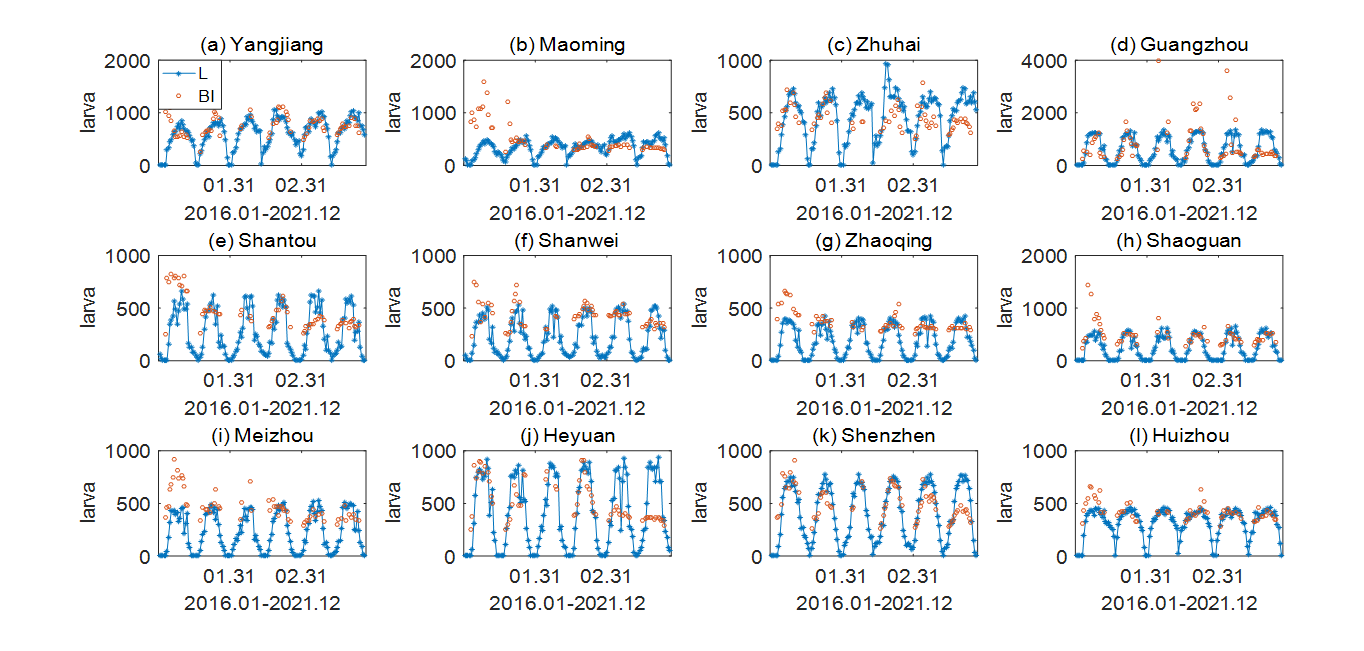

Supplement: S2 Fig — Blue asterisks represent fitted results, and the red circles represent the actual BI values. (TIF) [file pntd.0011247.s004.tif]

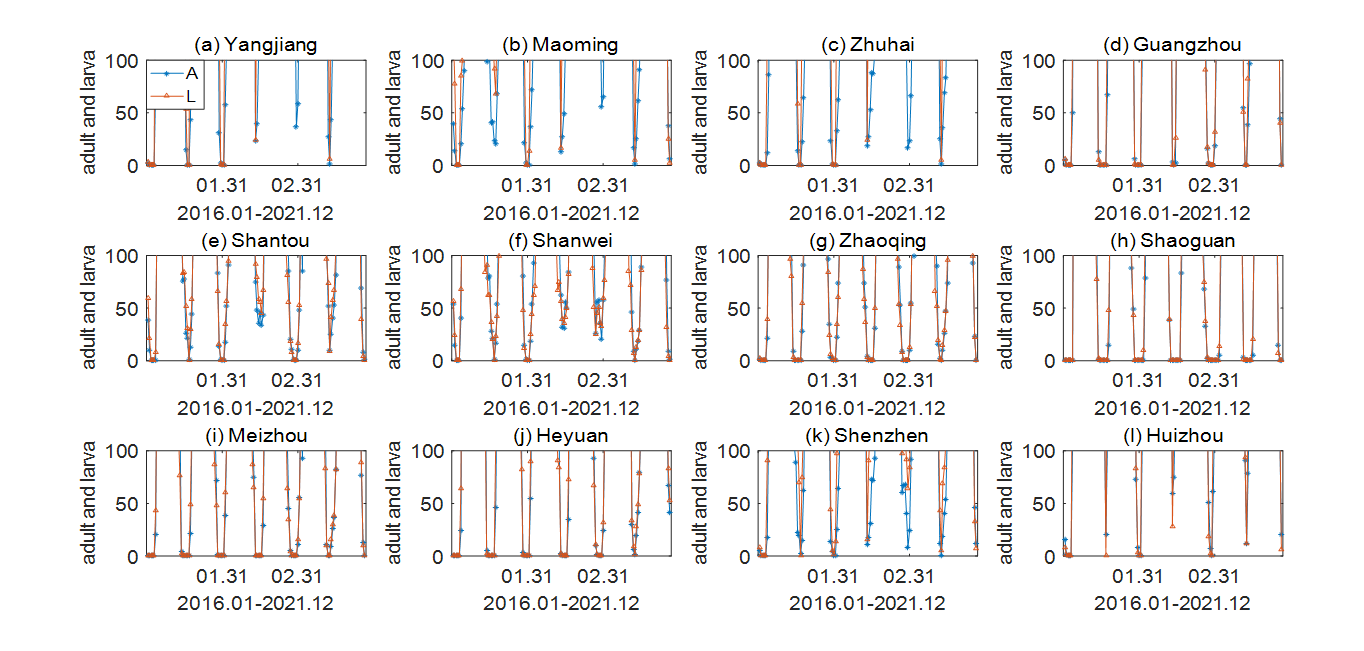

Supplement: S3 Fig — The overwintering of adult mosquitoes and larvae in the 12 municipalities from 2016 to 2021 by enlarging the bottom of the fitted results for larvae and adults in S1 and S2 Figs. Blue asterisks represent fitted results for adult mosquitoes and red triangles represent fitted results for larvae. (TIF) [file pntd.0011247.s005.tif]

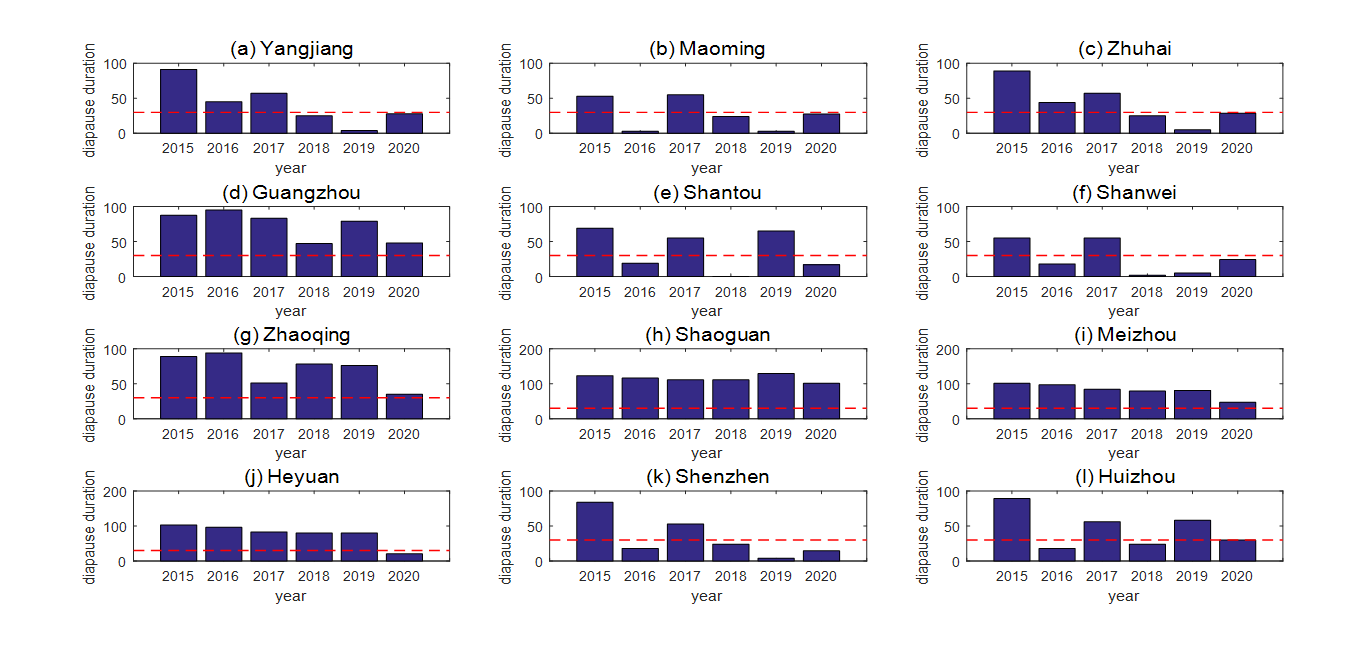

Supplement: S4 Fig — The red dashed line represents 30 days. (TIF) [file pntd.0011247.s006.tif]

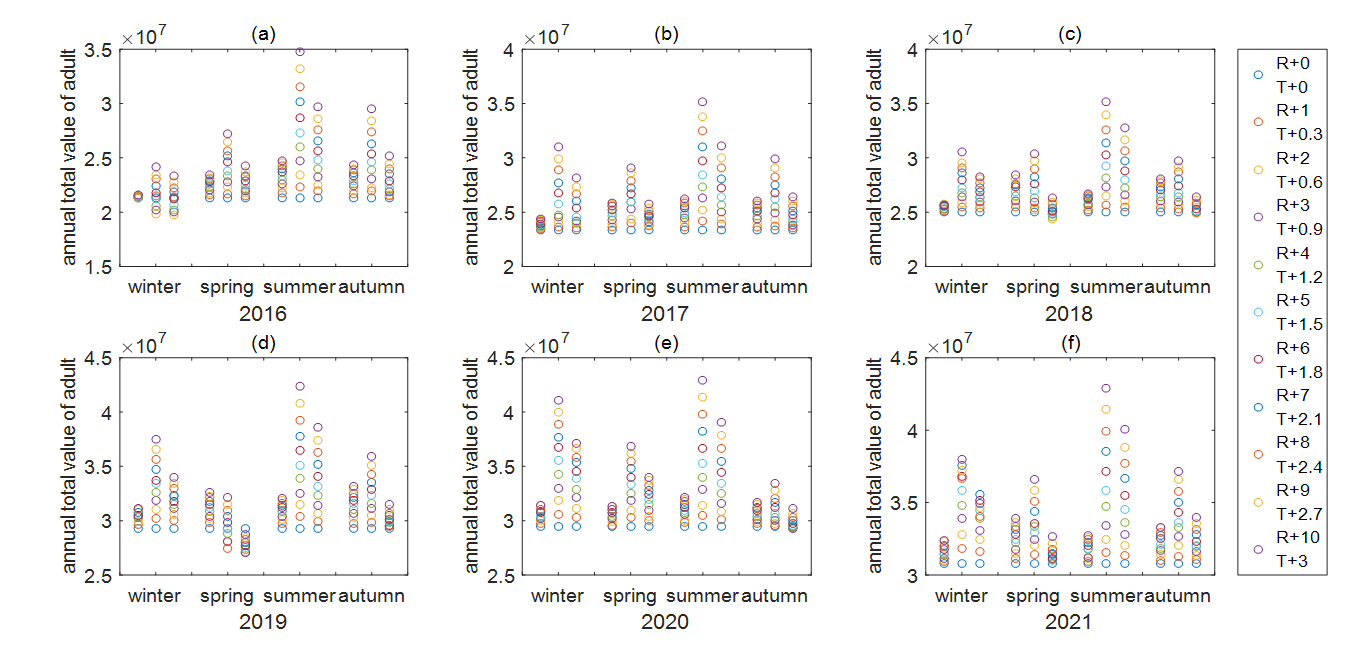

Supplement: S5 Fig — The three sets of data for each season in the sub-figure show the results of increasing seasonal rainfall, increasing both seasonal rainfall and seasonal temperature, and increasing seasonal temperature from left to right. The "winter" in the figure represents the winter of the previous year. For example, "winter" in the first subfigure represents the winter of 2015, including December 2015 and January and February 2016. The same is true for S6 and S7 Figs. (TIF) [file pntd.0011247.s007.tif]

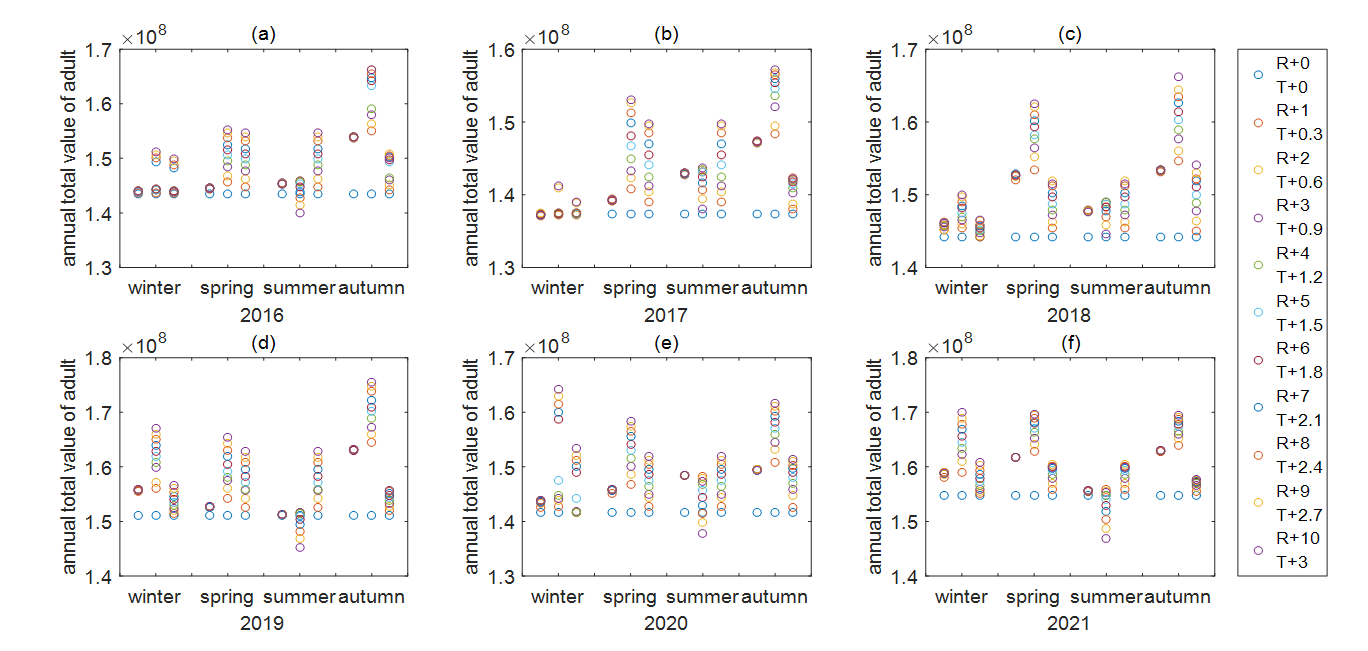

Supplement: S6 Fig — (TIF) [file pntd.0011247.s008.tif]

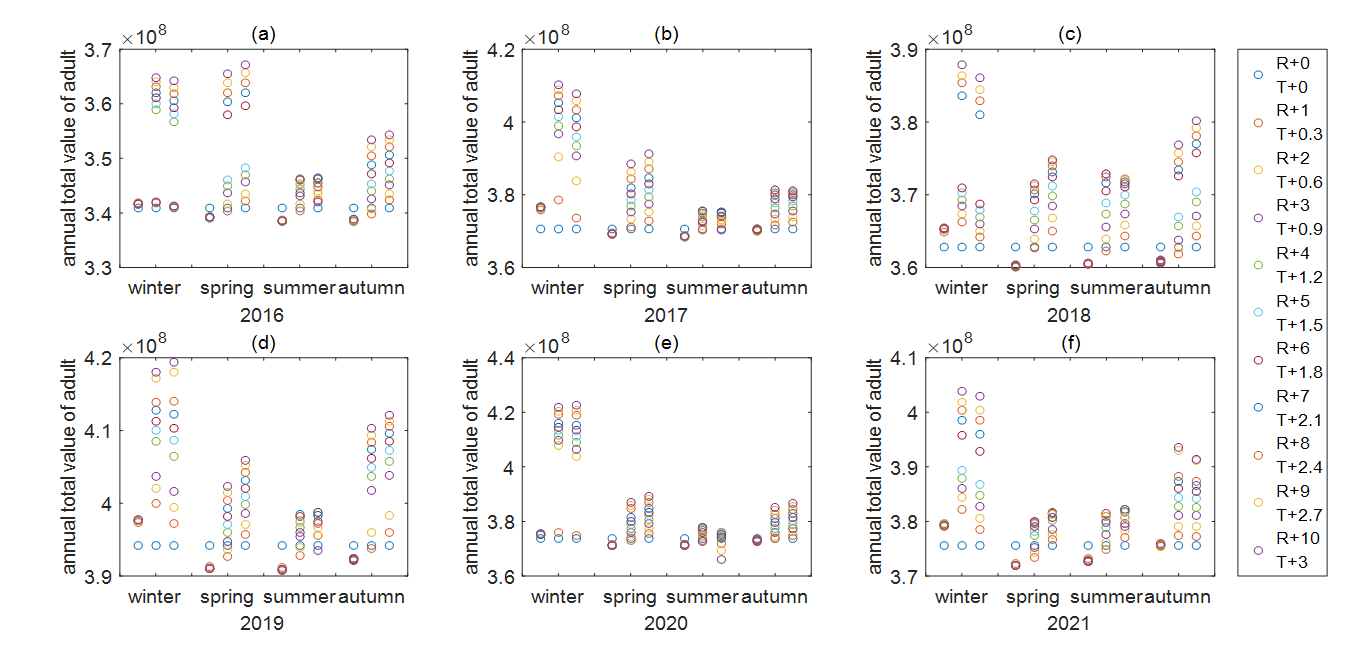

Supplement: S7 Fig — (TIF) [file pntd.0011247.s009.tif]

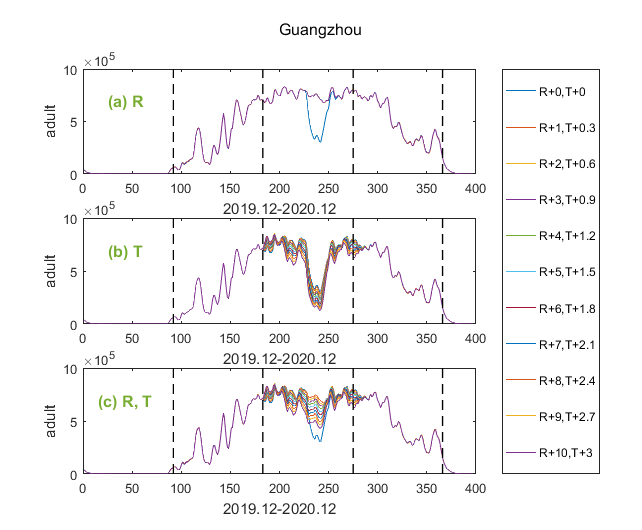

Supplement: S8 Fig — Changes in adult mosquito quantity when (a) increasing summer rainfall, (b) increasing summer temperature, and (c) simultaneously increasing summer rainfall and temperature. The black dashed line from left to right represents the end time of winter, spring, summer and autumn. The same is true for S9–S12 Figs. (TIF) [file pntd.0011247.s010.tif]

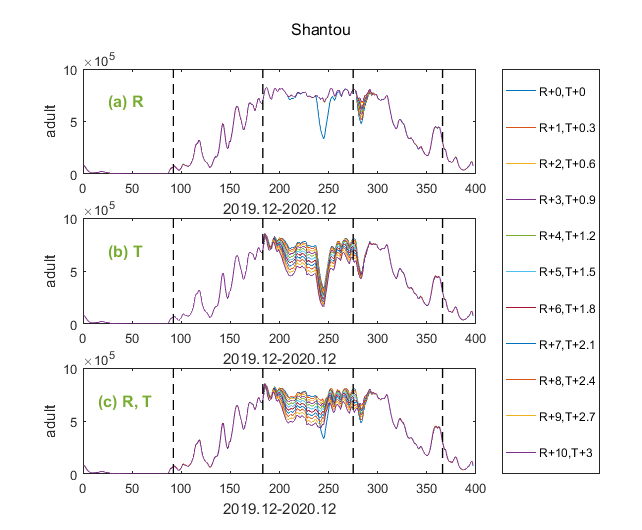

Supplement: S9 Fig — (TIF) [file pntd.0011247.s011.tif]

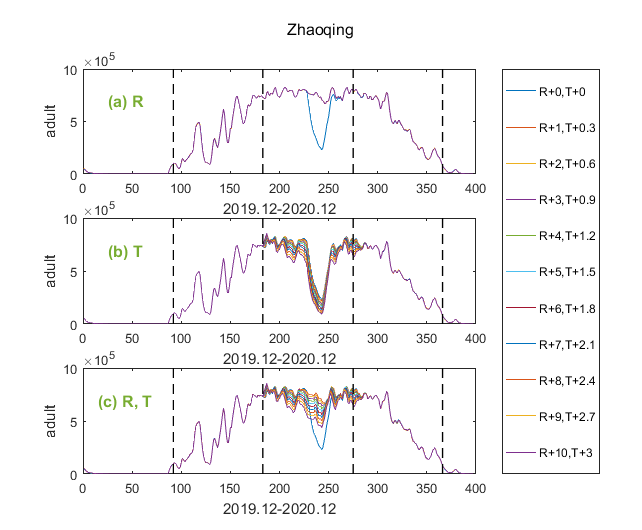

Supplement: S10 Fig — (TIF) [file pntd.0011247.s012.tif]

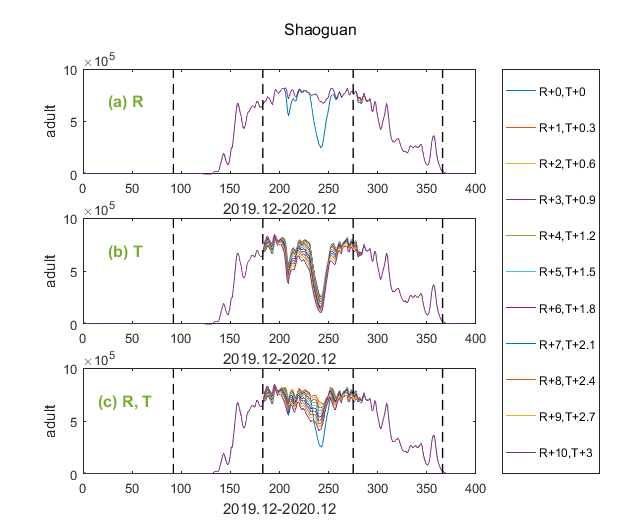

Supplement: S11 Fig — (TIF) [file pntd.0011247.s013.tif]

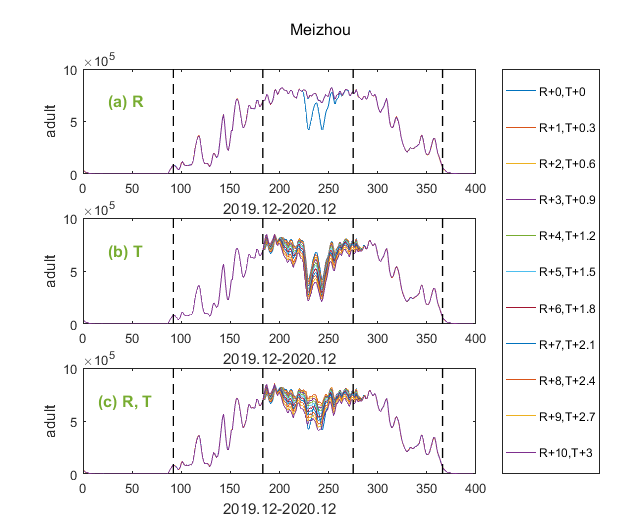

Supplement: S12 Fig — (TIF) [file pntd.0011247.s014.tif]

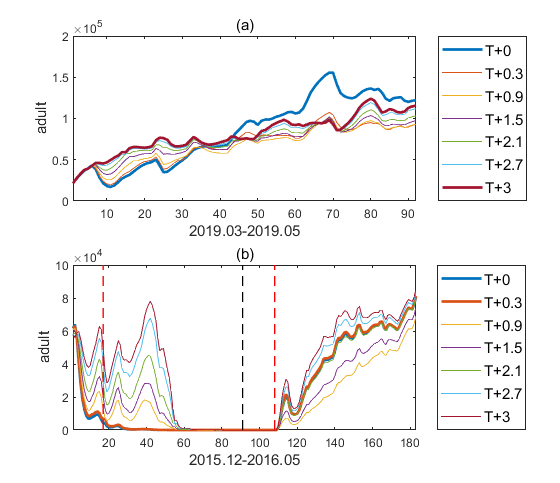

Supplement: S13 Fig — (a) The variation of adult mosquito quantity with increasing temperature in the spring of 2019; (b) The variation of adult mosquito quantity with increasing temperature in the winter of 2015, and the two red dashed lines in subgraph (b) represent, from left to right, the start and end of the diapause period (the longest diapause period in the experiment), and the black dashed line represents the winter end date. On the basis of the actual temperature in each season of each year, the temperature was increased by 3°C at intervals of 0.3. The curve in the figure represents the seasonal variation of the number of mosquitoes when the temperature was increased by 0.3. As the spring temperatures of 2018 and 2019 in Yangjiang are mostly between 18°C and 30°C, and the optimal survival temperatures for larvae, pupae and adult mosquitoes are 28°C, 30°C and 21°C respectively, the curves representing the changes in the number of mosquitoes will cross during the process of temperature increase, as shown in S13(A) Fig above, the crossing of thick and thin lines. As a result, the annual total adult mosquito quantity decreases and then rises with the increase of spring temperatures in 2018 and 2019 as shown in S5 Fig. (TIF) [file pntd.0011247.s015.tif]

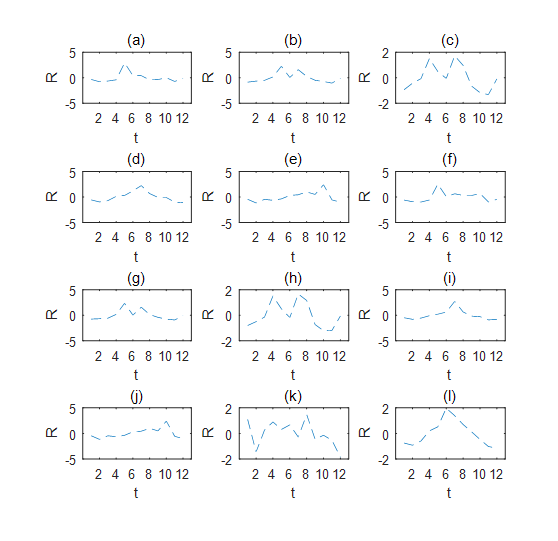

Supplement: S14 Fig — In the figure, the horizontal axis represents the month, and the vertical axis represents the average monthly rainfall after Z-Score standardization. Obviously, compared with these centroid distributions when classified into 6 categories (S15 Fig), subgraphs (a)-(e) do not include the situation when the annual distribution of rainfall is more uniform (S15(F) Fig). In addition, the rainfall peaks of distribution (i) and (l) in subfigures (f)-(l) are both in summer, and the peak height is about 2. According to the classification purpose (to find the annual distribution of typical climate), the two can obviously be classified into the same category. (TIF) [file pntd.0011247.s016.tif]

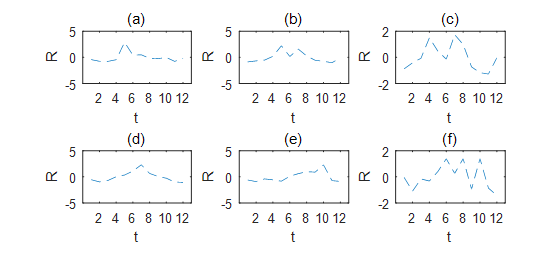

Supplement: S15 Fig — The horizontal axis represents the month, and the vertical axis represents the average monthly rainfall after Z-Score standardization. (TIF) [file pntd.0011247.s017.tif]

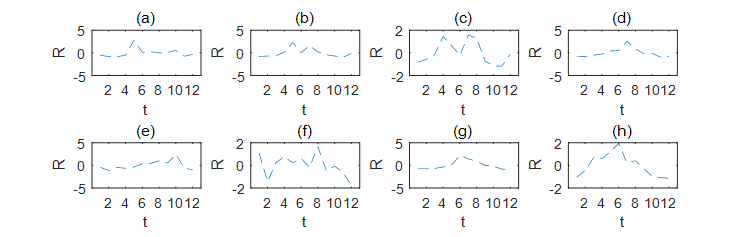

Supplement: S16 Fig — The horizontal axis represents the month, and the vertical axis represents the average monthly rainfall after Z-Score standardization. Compared with S15 Fig (these centroid distributions when classified into 6 categories), it is obvious that (d), (g) and (h) can also be classified into the same category, because the rainfall peaks of the three are in summer and the height is around 2. (TIF) [file pntd.0011247.s018.tif]
